# Identification and Validation of a Ferroptosis-Related Long Non-Coding RNA (FRlncRNA) Signature to Predict Survival Outcomes and the Immune Microenvironment in Patients With Clear Cell Renal Cell Carcinoma

**DOI:** 10.3389/fgene.2022.787884

**Published:** 2022-03-08

**Authors:** Zhongbao Zhou, Zhenpeng Yang, Yuanshan Cui, Shuai Lu, Yongjin Huang, Xuanyan Che, Liqing Yang, Yong Zhang

**Affiliations:** ^1^ Department of Urology, Beijing TianTan Hospital, Capital Medical University, Beijing, China; ^2^ Department of General Surgery, Beijing Shijitan Hospital, Capital Medical University, Beijing, China; ^3^ Department of Urology, The Affiliated Yantai Yuhuangding Hospital of Qingdao University, Yantai, China; ^4^ Department of Neurology, The Affiliated Yantai Yuhuangding Hospital of Qingdao University, Yantai, China

**Keywords:** ferroptosis, clear cell renal cell carcinoma, long non-coding RNAs, prognostic signature, overall survival, immune microenvironment

## Abstract

**Background:** The incidence of clear cell renal cell carcinoma (ccRCC) is increasing worldwide, contributing to 70–85% of kidney cancer cases. Ferroptosis is a novel type of programmed cell death and could predict prognoses in cancers. Here, we developed a ferroptosis-related long non-coding RNA (FRlncRNA) signature to improve the prognostic prediction of ccRCC.

**Methods:** The transcriptome profiles of FRlncRNAs and clinical data of ccRCC were obtained from The Cancer Genome Atlas and ICGC databases. Patients were randomly assigned to training cohorts, testing cohorts, and overall cohorts. The FRlncRNA signature was constructed by Lasso regression and Cox regression analysis, and Kaplan–Meier (K-M) analysis was used to access the prognosis of each group. The accuracy of this signature was evaluated by the receiver operating characteristic (ROC) curve. The visualization of functional enrichment was carried out by the gene set enrichment analysis (GSEA). Internal and external datasets were performed to verify the FRlncRNA signature.

**Results:** A FRlncRNA signature comprising eight lncRNAs (AL590094.1, LINC00460, LINC00944, AC024060.1, HOXB-AS4, LINC01615, EPB41L4A-DT, and LINC01550) was identified. Patients were divided into low- and high-risk groups according to the median risk score, in which the high-risk group owned a dramatical shorter survival time than that of the low-risk group. Through ROC analysis, it was found that this signature had a greater predictive capability than traditional evaluation methods. The risk score was an independent risk factor for overall survival suggested by multivariate Cox analysis (HR = 1.065, 95%CI = 1.036–1.095, and *p* < 0.001). We constructed a clinically predictive nomogram based on this signature and its clinical features, which is of accurate prediction about the survival rate of patients. The GSEA showed that primary pathways were the P53 signaling pathway and tumor necrosis factor–mediated signaling pathway. The major FRlncRNAs (LINC00460, LINC00944, LINC01550, and EPB41L4A-DT) were verified with the prognosis of ccRCC in the GEPIA and K-M Plotter databases. Their major target genes (BNIP3, RRM2, and GOT1) were closely related to the stage, grade, and survival outcomes of ccRCC by the validation of multiple databases. Additionally, we found two groups had a significant distinct pattern of immune function, immune checkpoint, and immune infiltration, which may lead to different survival benefits.

**Conclusions:** The FRlncRNA signature was accurate and act as reliable tools for predicting clinical outcomes and the immune microenvironment of patients with ccRCC, which may be molecular biomarkers and therapeutic targets.

## Introduction

Renal cell carcinoma (RCC) is a common solid tumor in kidney cancer, accounting for about 90% of renal malignancies (Ferlay et al., 2018; Compérat et al., 2019). The European Association of Urology (EAU) guidelines reported that the incidence of RCC has increased by approximately 2% per year in the past 2 decades (Bex et al., 2018). One of the most common pathological types in RCC is clear cell renal cell carcinoma (ccRCC), which accounts for about 75% of RCC (Ferlay et al., 2018). So it is very meaningful to identify molecular biomarkers to monitor the progression and early metastasis of ccRCC.

Ferroptosis is a novel type of programmed cell death, which is mainly characterized by lipid peroxidation (Chen et al., 2020). Ferroptosis is involved in the synthesis and metabolism of many molecules, including amino acids, polyunsaturated fatty acids, glutathione, phospholipids, and others (Stockwell et al., 2017; Xie and Guo, 2021). Additionally, ferroptosis can be inhibited by iron chelators, lipid peroxidation inhibitors, and reduction in intracellular polyunsaturated fatty acids (Dixon et al., 2015). Valashedi et al. reported that ferroptosis can inhibit tumor formation and progression, which may be beneficial in the treatment of cancer (Valashedi et al., 2021). The correlation between the expression of ferroptosis-related genes (FRGs) and tumorigenesis has not been deeply investigated.

Long noncoding RNAs (lncRNAs) have been shown to participate in various considerable biological processes, such as cell proliferation and differentiation, gene regulation and translation, RNA splicing, regulation of microRNAs, and protein folding (Panni et al., 2020). The mechanism of ferroptosis during cancer development was rarely reported. Ferroptosis regulated by lncRNAs was known to participate in the various progression stages of ccRCC, such as invasion, metastasis, prognosis, and chemoresistance. The limitation of multiple studies is that they only target the single or a few lncRNAs investigated for ccRCC (Ju et al., 2020; Zhu et al., 2020; Yang et al., 2021). To date, novel biomarkers have not been reported to be explored *via* the lncRNAs’ expression profiles of The Cancer Genome Atlas (TCGA) database to predict the prognosis of ccRCC. Therefore, we developed a ferroptosis-related long non-coding RNA (FRlncRNA) signature using TCGA database to find new biomarkers to predict prognosis of ccRCC.

## Materials and Methods

### Datasets and Sample Extraction

The FPKM-RNA sequence and clinical information of ccRCC were downloaded from the TCGA-KIRC data portal (https://portal.gdc.cancer.gov/), where it contained a total of 537 ccRCC tissues and 72 adjacent tissues. The clinical information mainly included age, gender, clinical stage, T stage, N stage, M stage, survival status, survival time, and survival prognosis. The exclusion criteria were as follows: 1) The pathological diagnosis did not meet ccRCC; 2) The RNA sequence and clinical data were incomplete; and 3) The follow-up time did not exceed 30 days. Subsequently, the expression data of these RNAs were sorted, annotated, and then assigned to protein-coding genes and lncRNAs using the Ensembl human genome browser (http://asia.ensembl.org/info/data/index.html) and Perl program. The extracted data were normalized and processed by log2 transformation. The expression data of 89 ccRCC patients from the ICGC database (https://dcc.icgc.org/analysis) were obtained for the external validation of the FRlncRNA signature. The “limma” package in R software was utilized to correct the transcriptome data we have downloaded.

### Screening of FRlncRNAs and Differentially Expressed Genes

The FerrDb database (http://www.zhounan.org/ferrdb/legacy/index.html) was used to obtain the FRG dataset, containing a total of 214 FRGs (Supplementary data) where 203 FRGs were found in the TCGA dataset. Among them, 62 FRGs were differentially expressed in ccRCC. The relation between FRGs and lncRNAs was analyzed by the Pearson correlation analysis. LncRNAs would be included in this study when the square of the correlation coefficient (*R* ^2^) was greater than 0.3, and concomitantly, the *p*-value was lower than 0.001. Finally, the “limma” package in R software was applied to extract a total of 1,669 FRlncRNAs (Supplementary data).

### Identification of the Prognostic FRlncRNA Signature

To establish an effective prognostic prediction model, we randomly divided 537 ccRCC patients into training cohorts and testing cohorts in a 1:1 ratio. Finally, 243 people were included in the training cohorts, and 264 people were included in the testing cohorts, according to the exclusion criteria. The detailed patient grouping process is shown in Supplementary Figure S1. The basic characteristics of each group are showed in Table 1 (Details in Supplementary data). The FRlncRNA signature was built according to the training cohorts, and the capability of predicting prognosis was evaluated *via* the testing cohorts, overall cohorts, and ICGC cohorts. The prognostic capability of FRlncRNAs in the training cohorts was assessed by the univariate Cox regression analysis. If *p* < 0.05, it would be included in the least absolute shrinkage and selection operator (Lasso) regression using the “glmnet” package in R software to avoid overfitting. The risk score of each patient, established by incorporating the Lasso regression into the multivariate Cox regression analysis, was calculated according to the function, ∑^n^_i=1_β_i_∗ (expression of lncRNA_i_), where β represented the regression coefficient. Patients were classed into high- and low-risk groups based on the median risk score, and the survival rate between two groups was compared using the log-rank test.

**TABLE 1:** The characteristics of ccRCC patients included in this study.

Variable		Overall cohorts (n = 507)	Training cohorts (n = 243)	Testing cohorts (n = 264)	ICGC cohorts (n = 89)
Age (year, Mean ± SD)		60.26 ± 12.08	60.21 ± 12.46	60.31 ± 11.72	60.48 ± 10.06
Gender (n, %)	Male	333 (65.7)	163 (67.1)	170 (64.4)	50 (56.2)
	Female	174 (34.3)	80 (32.9)	94 (35.6)	39 (43.8)
Stage (n, %)	Stage I	253 (49.9)	116 (47.7)	137 (51.9)	0 (0.0)
	Stage II	53 (10.5)	28 (11.5)	25 (9.5)	0 (0.0)
	Stage III	116 (22.9)	59 (24.3)	57 (21.6)	0 (0.0)
	Stage IV	82 (16.2)	39 (16.0)	43 (16.3)	0 (0.0)
	Unknown	3 (0.5)	1 (0.5)	2 (0.7)	89 (100.0)
T stage (n, %)	T1	259 (51.1)	119 (49.0)	140 (53.0)	51 (57.3)
	T2	65 (12.8)	34 (14.0)	31 (11.7)	9 (10.2)
	T3	172 (33.9)	84 (34.6)	88 (33.3)	27 (30.3)
	T4	11 (2.2)	6 (2.4)	5 (2.0)	2 (2.2)
N stage (n, %)	N0	225 (44.4)	90 (37.0)	135 (51.1)	40 (44.9)
	N1	16 (3.2)	10 (4.1)	6 (2.3)	0
	NX	266 (52.4)	143 (58.9)	123 (46.6)	49 (55.1)
M stage (n, %)	M0	401 (79.1)	198 (81.5)	203 (76.9)	35 (39.3)
	M1	78 (15.4)	38 (15.6)	40 (15.2)	4 (4.5)
	MX	26 (5.1)	7 (2.9)	19 (7.2)	50 (56.2)
	Unknown	2 (0.4)	0 (0.0)	2 (0.7)	0 (0.0)
Survival status (n, %)	Alive	162 (34.0)	77 (31.7)	85 (32.2)	60 (67.4)
	Dead	345 (66.0)	166 (68.3)	179 (67.8)	29 (32.6)
Survival years (Mean ± SD)		3.25 ± 2.18	3.00 ± 2.03	3.48 ± 2.29	4.17 ± 1.69

SD, standard deviation.

The risk score of each patient in the test cohorts, overall cohorts, and ICGC cohorts was calculated using the same way to confirm the stability of the established model. The survival outcomes of each cohort were analyzed by the Kaplan–Meier (K-M) survival curve. The “ROC package” in R software was employed to analyze the specificity and sensitivity of the established model based on the ROC curve and its area under the curve (AUC) value.

### Construction and Evaluation of the Prognostic Nomogram

A prognostic nomogram according to the aforementioned risk score and traditional prognosis-related clinical variables (age, grade, and stage) was established to statistically predict the prognosis of ccRCC patients. Subsequently, the reliability and accuracy of the nomogram were evaluated *via* the concordance index (C-index), calibration curve, and ROC curve. The basic characteristics of patients were included into the multivariate Cox regression analysis to determine whether the risk score was an independent predictor of prognosis.

### Functional Enrichment Analysis

The gene set enrichment analysis (GSEA) software was employed to perform the explanation of the functional enrichment of these FRlncRNAs. The pathway components of the high-risk group and the differences of the pathway activity and expression patterns, which were downloaded from MSigDB and GSEA 4.1.0, were analyzed in the pathway analysis dataset including c2. cp.kegg. v7.4. symbols and c5. go.bp. v7.4. symbols. A two-tailed *p*-value less than 0.05 was considered to be significant. To clarify how target genes of these FRlncRNAs participated in the development of ccRCC, we performed a functional analysis of related FRGs. The “clusterProfiler” package (Yu et al., 2012) and “org.Hs.eg.db” package in R software were used for functional enrichment of target genes based on the Kyoto Encyclopedia of Genes and Genomes (KEGG) signaling pathway and gene ontology (GO) enrichment analysis. The cut-off criterion was set as *p* less than 0.05 and a q value more than 0.05.

### Interaction Network Construction and Verification

When the co-expression coefficient was greater than 0.3 and the *p*-value was less than 0.001 using the “limma” package, we believed that there was a good correlation between FRlncRNAs and FRGs. Cytoscape 3.6.0 software was used to visualize the network between eight FRlncRNAs and related FRGs. After that, we further identified the major FRlncRNAs and related FRGs by searching the relevant literature and multiple databases. We also explored the ceRNA network of major FRlncRNAs. The gene expression profiling interactive analysis (GEPIA, http://gepia.cancer-pku.cn/) contained RNA-seq and clinical data compiled by TCGA and GTEx after standardized analysis. The UALCAN online database (http://ualcan.path.uab.edu/index.html) reported the differences of the target gene expression between normal tissues and ccRCC-graded tissues. The K-M Plotter database (http://kmplot.com/analysis/) included data on the correlation between the gene expression and prognostic data of 530 patients with ccRCC. The expression levels of the major FRlncRNAs-FRGs and the prognostic correlation were verified in the above three databases.

### RNA Extraction, Reverse Transcription, and Quantitative Real-time PCR (qRT-PCR)

A total RNA extraction micro kit (RNT411-03, Mabio, Guangdong, China; http://www.mabiotech.cn/en/list-643-1.html) was used to extract the total RNA from tumor tissues and normal tissues based on the operation instructions, and then, a spectrophotometer was employed to detect the concentration and check the quality of total RNA. Then, a cDNA synthesis kit with random primers (AG11711, AG, Changsha, China; https://agbio.com.cn/product/evo-m-mlv-rt-kit-with-gdna-clean-for-qpcr-ii/?v=b838b393d55f) was used in a 20 μ1 reaction volume with 1 μg total RNA for cDNA synthesis. The mRNA expression was detected by the Script SYBR Green PCR kit (AG11702, AG, Changsha, China; https://agbio.com.cn/product/sybr-green-premix-pro-taq-hs-qpcr-kit-ii/?v=b838b393d55f) *via* an ABI7900HT Fast Real-time PCR machine, with the following conditions: pre-denaturation at 95°C for 30 s, followed by 40 cycles of denaturation at 95°C for 5 s, and annealing and extension at 60°C for 30 s. GAPDH was the internal control for mRNA, and the 2-ΔΔCt function was used to calculate the gene expression level (DiMagno, 2007). QRT-PCR primer sequences used in our experiments are listed as follows: GAPDH forward primer (F): 5′-TGA​CTT​CAA​CAG​CGA​CAC​CCA-3’; GAPDH reverse primer (R): 5′-CAC​CCT​GTT​GCT​GTA​GCC​AAA-3’; LINC00460 F: 5′-TAA​ACC​TAG​GGG​CCG​GTC​G-3’; LINC00460 R: 5′-AAC​GGT​CCA​GAG​CAG​ACA​AAA-3’; LINC00944 F: 5′-AGA​CGC​ACA​TCA​GGA​AGA​CAG-3’; LINC00944 R: 5′-TGA​GTT​ACA​GGG​ACC​GAA​GC-3’; LINC01550 F: 5′-GGT​GCA​GTC​TCC​TCA​GAA​CTA​C-3’; LINC01550 R: 5′-GGG​AGA​GGG​AGA​ACG​ACT​GT-3’; EPB41L4A-DT F: 5′-CGG​AGC​AGG​TGC​AAT​CTG​T-3’; and EPB41L4A-DT R: 5′-TCA​AAA​CTA​CGT​CTG​ATG​CCA​AA-3’.

### Statistical Analysis

The two-tailed Student’s t-test was calculated for the difference within groups or among groups. Categorical variables were presented as proportions, and the chi-square test was used for the comparisons between groups. Multivariate or univariate Cox proportional hazard regression analysis was calculated for evaluating the prognostic significance. The log-rank test and K-M curve assessed the prognostic results. R software (version 3.6.0) was used to draw the heatmap, GSEA, survivorship curve, ROC curve, nomogram, and calibration plot. A two-tailed *p*-value less than or equal to 0.05 was considered as the statistical significance.

## Results

### Construction and Verification of the FRlncRNA Signature

**FIGURE 1:**
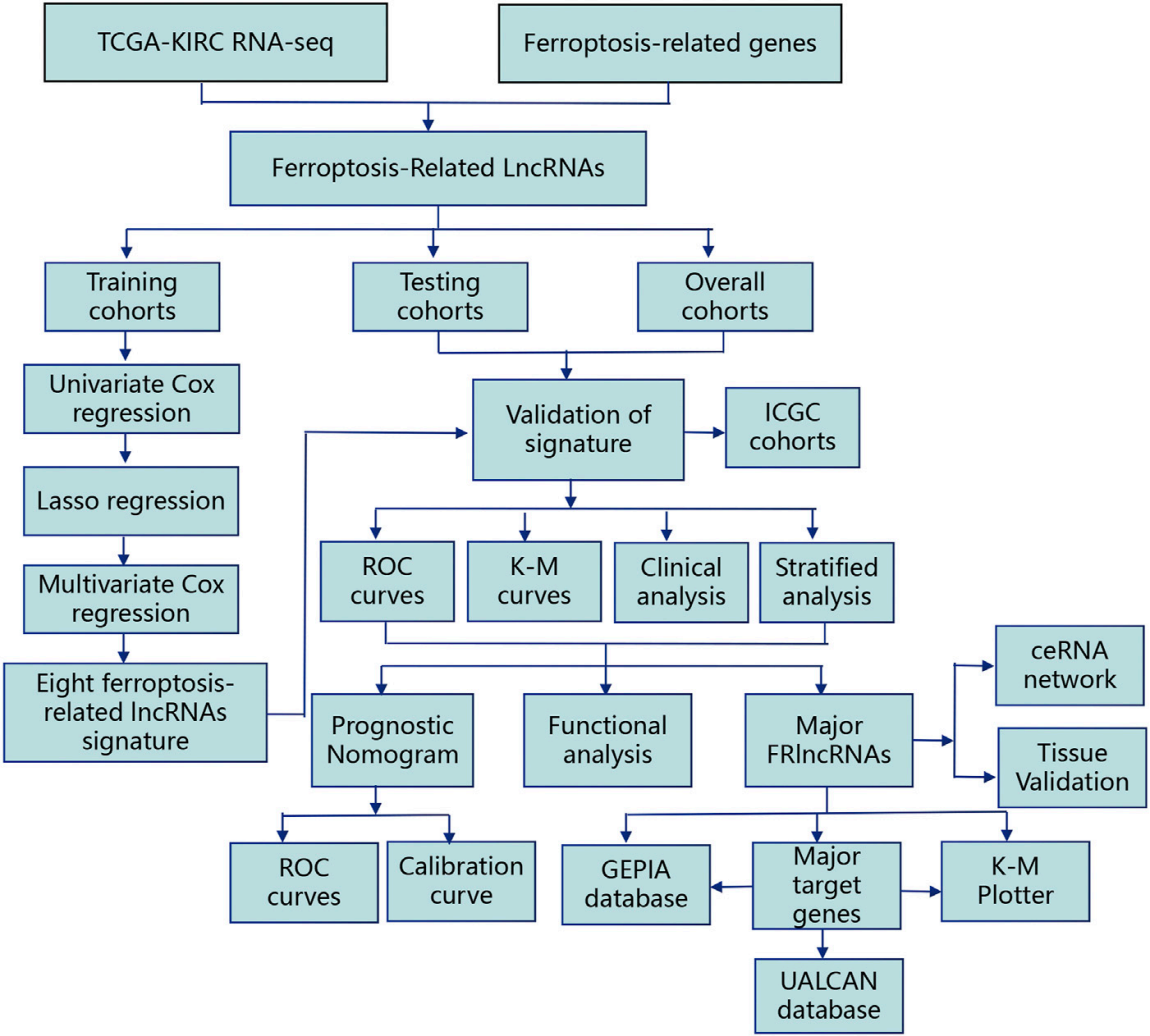
Analysis flow chart of this research.

**FIGURE 2:**
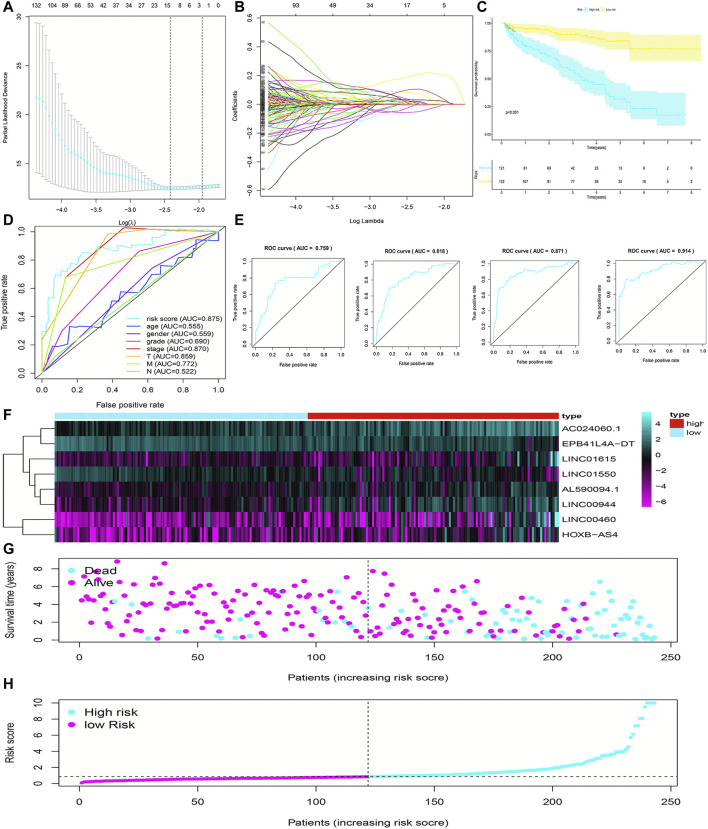
Construction and evaluation of the FRlncRNA signature in training cohorts. The LASSO regression analysis was performed to avoid overfitting in training cohorts after univariate Cox regression analysis. LASSO coefficient values and vertical dashed lines were calculated at the best log(lambda) value **(A)** and LASSO coefficient profiles **(B)** of the prognostic-related lncRNAs were displayed. **(C)** K-M curves showed that the high-risk group had worse survival probability than the low-risk group in the training cohorts. **(D)** ROC curves for this signature and their AUC values in training cohorts. **(E)** ROC curves and their AUC values represented 1-, 3-, 5-, and 10-year predictions in training cohorts. **(F)** Heatmap of the eight FRlncRNA expression profiles showed the expression of FRlncRNAs in the high-risk and low-risk groups in training cohorts. **(G)** Scatter plot showed the correlation between the survival status and risk score of ccRCC patients. **(H)** Risk score distribution plot showed the distribution of high-risk and low-risk ccRCC patients.

The flowchart of this work is showed in Figure 1. The univariate Cox regression analysis was used to analyze the expression of FRlncRNAs in the training cohorts. A total of 678 lncRNAs were found to be closely related with the prognosis of ccRCC. High overfitting of these prognostic-related lncRNAs were eliminated by Lasso Cox analysis, and 15 FRlncRNAs were identified (Figure 2A; Supplementary data). Then, multivariate Cox regression analysis extracted a prognostic signature containing eight FRlncRNAs and their coefficients (Supplementary Table S1; Figure 2B), calculated with the following formula: Risk score = (0.190*AL590094.1) + (0.047*LINC00460) + (0.204* LINC00944) + (0.168*AC024060.1) + (0.116*HOXB-AS4) + (0.048* LINC01615)—(0.134* EPB41L4A-DT)—(0.345* LINC01550). Based on the hazard ratio (HR) score gained by the multivariate Cox regression analysis, AC024060.1, AL590094.1, HOXB-AS4, LINC00460, LINC00944, and LINC01615, whose HRs were greater than 1, were risk factors, but EPB41L4A-DT and LINC01550, whose HRs were less than 1, were protective factors (Supplementary Table S1).

**FIGURE 3:**
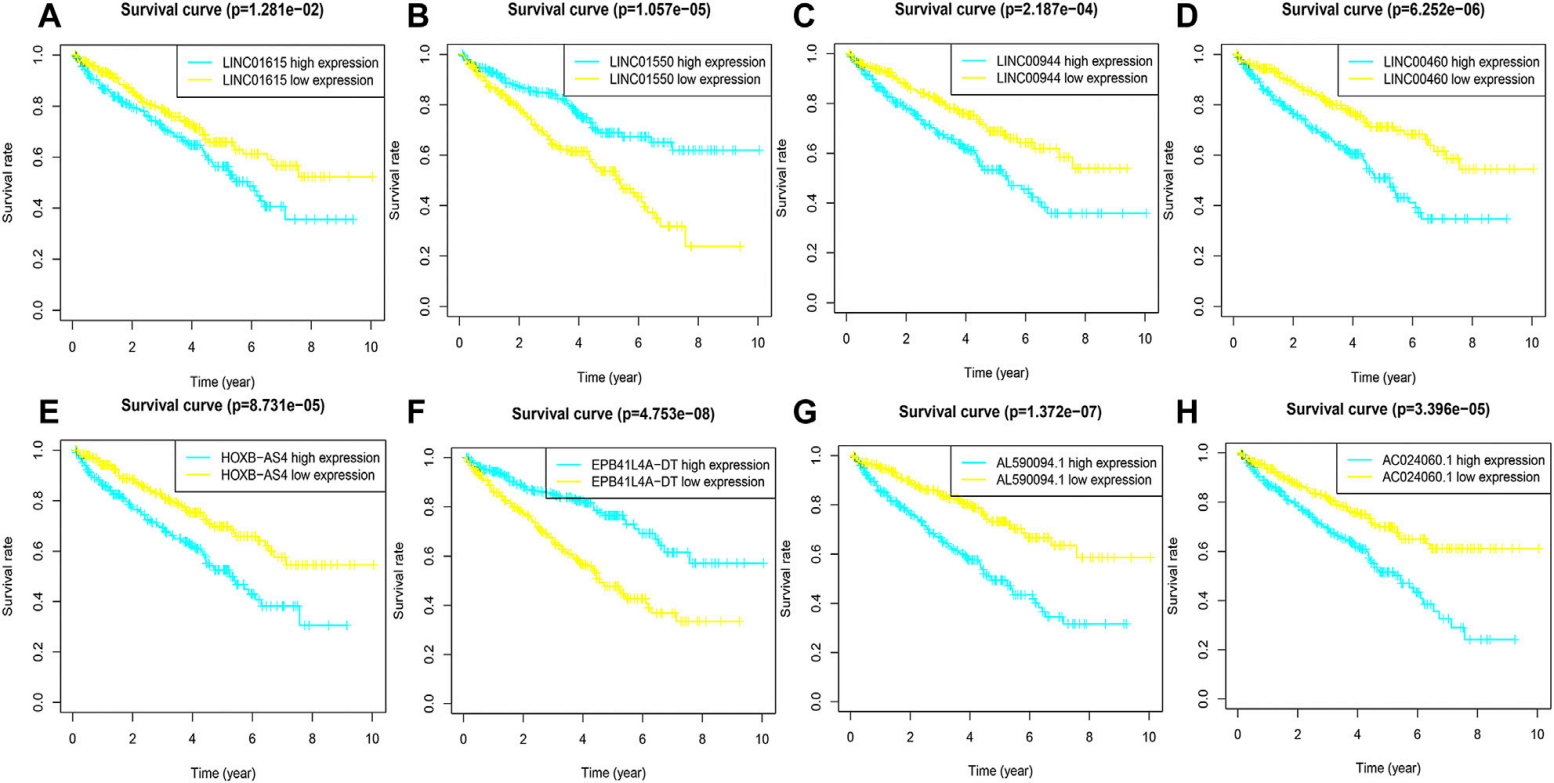
K-M curves of eight FRlncRNAs in the prognostic signature. **(A-H)** The K-M survival curves of AC024060.1, AL590094.1, HOXB-AS4, LINC00460, LINC00944, and LINC01615 showed the high expression group had worse OS than the low expression group in the training cohorts (*p* < 0.05) and the K-M curves of EPB41L4A-DT and LINC01550 showed the high expression group had better OS than the low expression group in the training cohorts (*p* < 0.05).

To evaluate the sensitivity and stability of the prognostic risk score, the training cohorts were divided into the low-risk group (122 cases) and the high-risk group (121 cases) based on the median of risk scores (0.86). The results of the K-M curve showed that the survival ability of patients in the high-risk group was significantly lower than that in the low-risk group (*p* < 0.001) (Figure 2C). The accuracy of the prognostic signature was evaluated by the ROC curve, and the AUC value was 0.875 (Figure 2D). The AUC values of 1, 3, 5, and 10 years of overall survival (OS) were 0.759, 0.818, 0.871, and 0.914, respectively (Figure 2E). The heatmap showed remarkable differences in the expression of eight FRlncRNAs between the high-risk group and the low-risk group (Figure 2F). The scatter plot indicated that ccRCC patients with a high risk score had a lower survival rate than those with a low-risk score (Figure 2G). Moreover, the distribution map of the risk score was consistent with the categorization of patient groups (Figure 2H). The prognostic effect of eight FRlncRNAs evaluated by K-M curves displayed that higher expressions of AC024060.1, AL590094.1, HOXB-AS4, LINC00460, LINC00944, and LINC01615 and lower expressions of EPB41L4A-DT and LINC01550 were linked to inferior OS (*p* < 0.01) (Figures 3A–H). The prognostic risk-related model we constructed exhibited a good stability and sensitivity in predicting the OS of ccRCC patients.

### Validation of the FRlncRNA Signature

**FIGURE 4:**
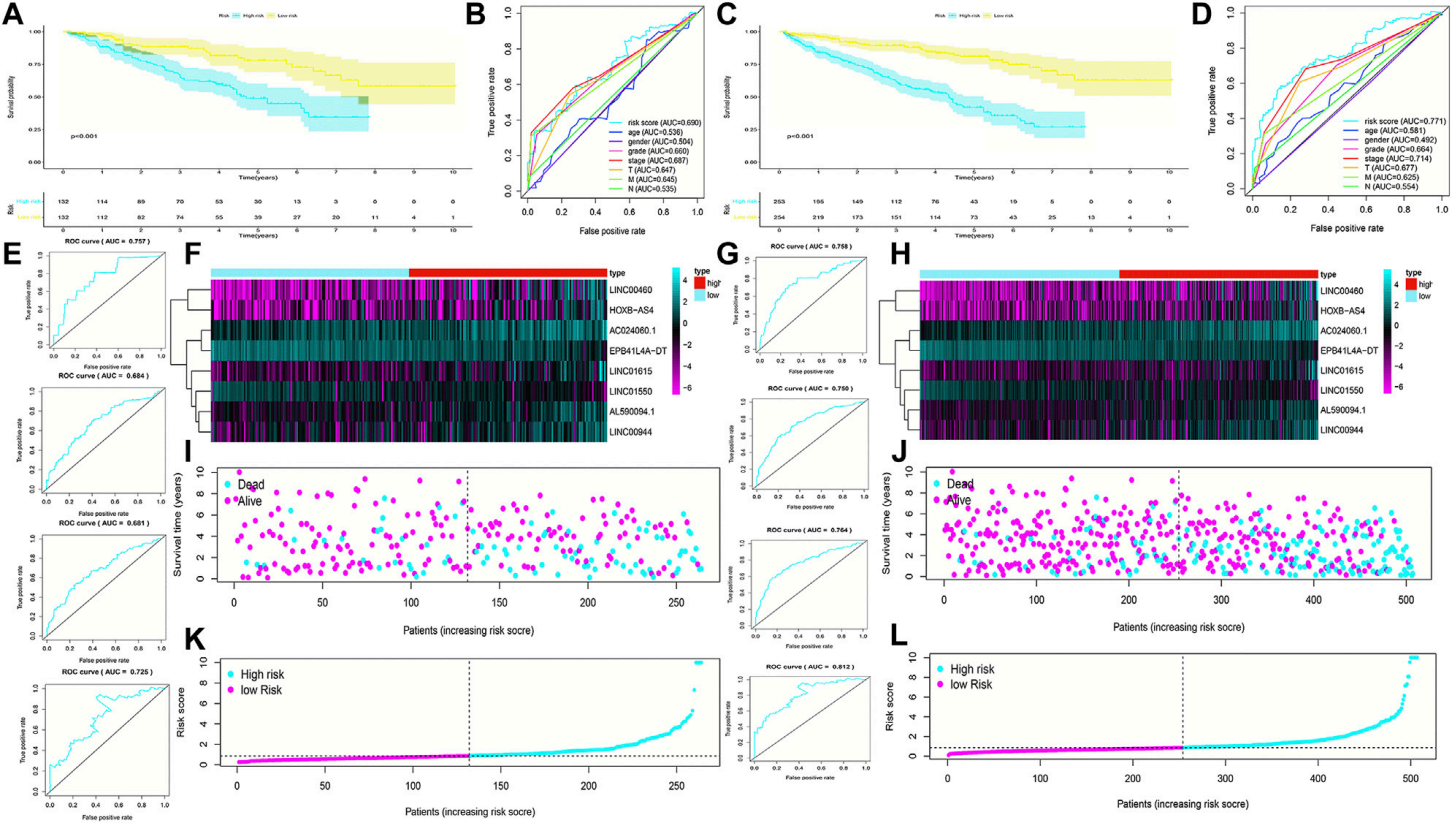
Validation of the prognostic signature for ccRCC patients in testing cohorts and overall cohorts. K-M curves showed that the high-risk group had worse OS than the low-risk group in the testing cohorts **(A)** and overall cohorts **(C)**. ROC curves for the prognostic signature and their AUC values in the testing cohorts **(B)** and overall cohorts **(D)**. ROC curves and their AUC values represented 1-, 3-, 5-, and 10-year predictions in the testing cohorts **(E)** and overall cohorts **(G)**. Heatmap of eight FRlncRNA expression profiles showed the expression of FRlncRNAs in high-risk and low-risk groups in the testing cohorts **(F)** and overall cohorts **(H)**. Scatter plot showed the outcomes between the survival status and risk score of ccRCC patients in high- and low-risk groups in the testing cohorts **(I)** and overall cohorts **(J)**. Risk score distribution plot showed the distribution of high- and low-risk ccRCC patients in the testing cohorts **(K)** and overall cohorts **(L)**.

To validate the predictive capacity of the FRlncRNA signature, risk scores of patients were calculated in the testing cohorts and overall cohorts, and patients were classified into the low-risk group and the high-risk group based on the median of risk scores (0.85 and 0.86, respectively). The OS in the testing cohorts (*p* < 0.001) (Figure 4A) and overall cohorts (*p* < 0.001) (Figure 4C) were analyzed by K-M curves, demonstrating that these results were in line with the training cohorts. The ROC curves of testing cohorts (AUC = 0.690) (Figure 4B) and overall cohorts (AUC = 0.771) (Figure 4D) illustrated that the FRlncRNA signature has an accurate predictive capability about the OS of ccRCC patients, which was further validated by ROC time curves and their AUC values, such as the AUC value in testing cohorts, 1-year AUC = 0.757, 3-year AUC = 0.684, 5-year AUC = 0.681, and 10-year AUC = 0.725 (Figure 4E) and the AUC value in overall cohorts (1-year AUC = 0.758, 3-year AUC = 0.750, 5-year AUC = 0.764, and 10-year AUC = 0.812) (Figure 4G). The consistent expression profiles of eight FRlncRNAs in the training cohorts are shown in the heatmap (Figures 4F,H). A lower survival rate was observed in the high-risk group than the low-risk group, and distribution maps of the risk score validated a higher risk score in the high-risk group (Figures 4I–L). In addition, ICGC cohorts were used to evaluate the constructed model, which showed a good ability to predict the survival rate of patients with ccRCC (Supplementary Figure 2A–E). These results showed that the FRlncRNA signature can be a good indicator in predicting the prognosis of patients compared with other existing signatures reported in recent studies (Supplementary Table S2) (Canxuan and Dan, 2021; Hong et al., 2021; Ma et al., 2021; Xing et al., 2021; Yu et al., 2021; Zheng et al., 2021). Taken together, our data implied that the FRlncRNA signature showed a stable prognostic-predictive ability.

### Stratified Analysis of Prognosis-Related Clinicopathological Characteristics

**FIGURE 5:**
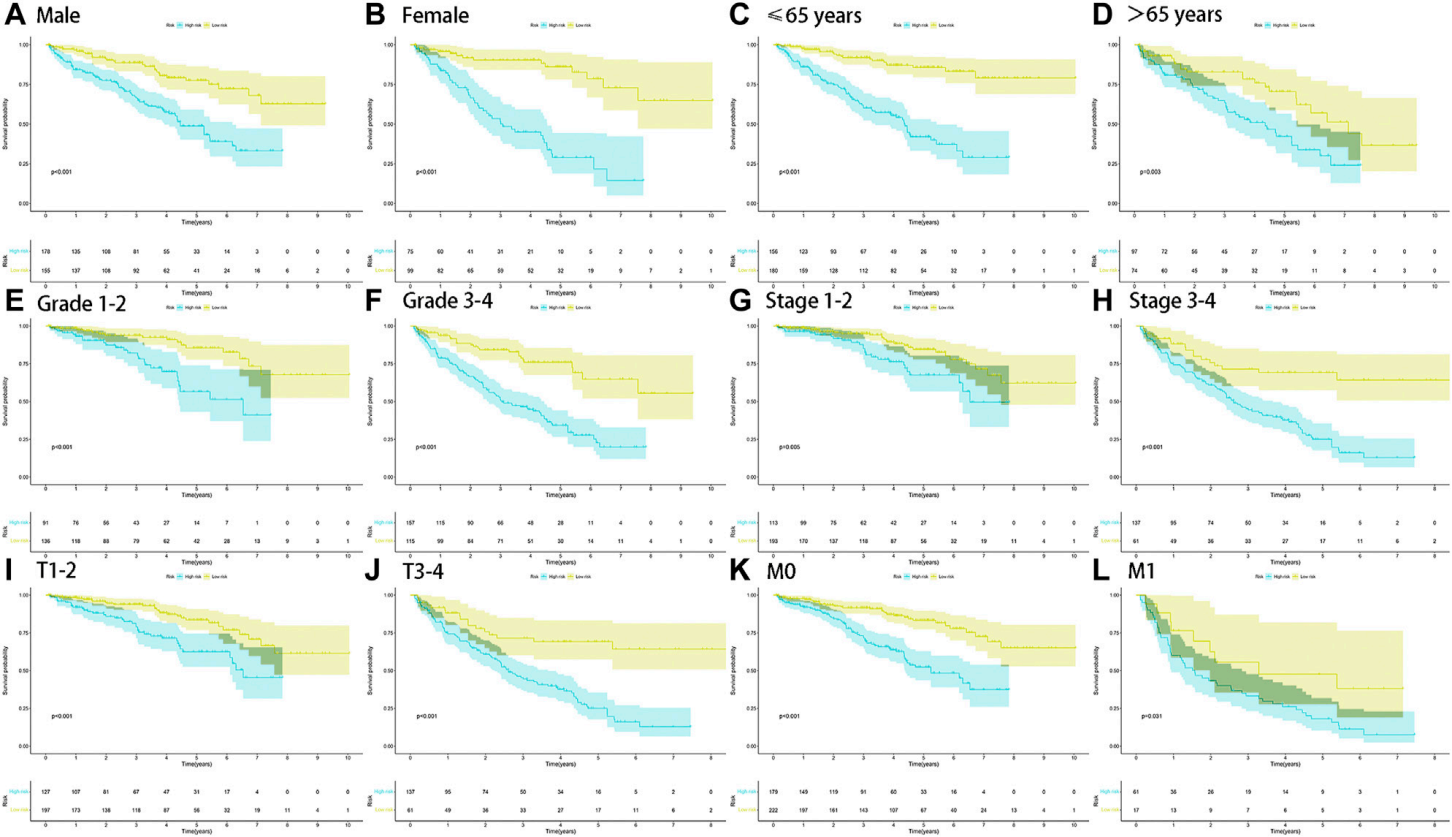
Survival outcomes of ccRCC patients stratified by various clinicopathological features. K-M curves showed the survival outcomes of high- and low-risk ccRCC patients stratified according to the gender (MALE vs. FEMALE) **(A,B)**, age (≤65 years vs. >65 years) **(C,D)**, grade (grade I-II vs. grade III-IV) **(E,F)**, stage (stage I-II vs. stage III-IV) **(G,H)**, T stage (T1-2 vs. T3-4) **(I,J)**, and M stage (M0 vs. M1) **(K,L)**, respectively (all *p* < 0.05).

The stratified analysis of clinicopathological characteristics was performed to assess the predictive ability of the FRlncRNA signature and the stability of its OS prediction in the high-risk and low-risk groups including gender (female and male), age (≤65 years old and >65 years old), grade (I-II and III-IV), stage (I-II and III-IV stage), T stage (T1-2 stage and T3-4 stage), and M stage (M0 stage and M1 stage). The results of the K–M curve in different clinical characteristics suggested that the OS of the high-risk group was worse than that of the low-risk group (*p* < 0.01) (Figures 5A–L).

### Construction and Evaluation of the Prognostic Nomogram

**FIGURE 6:**
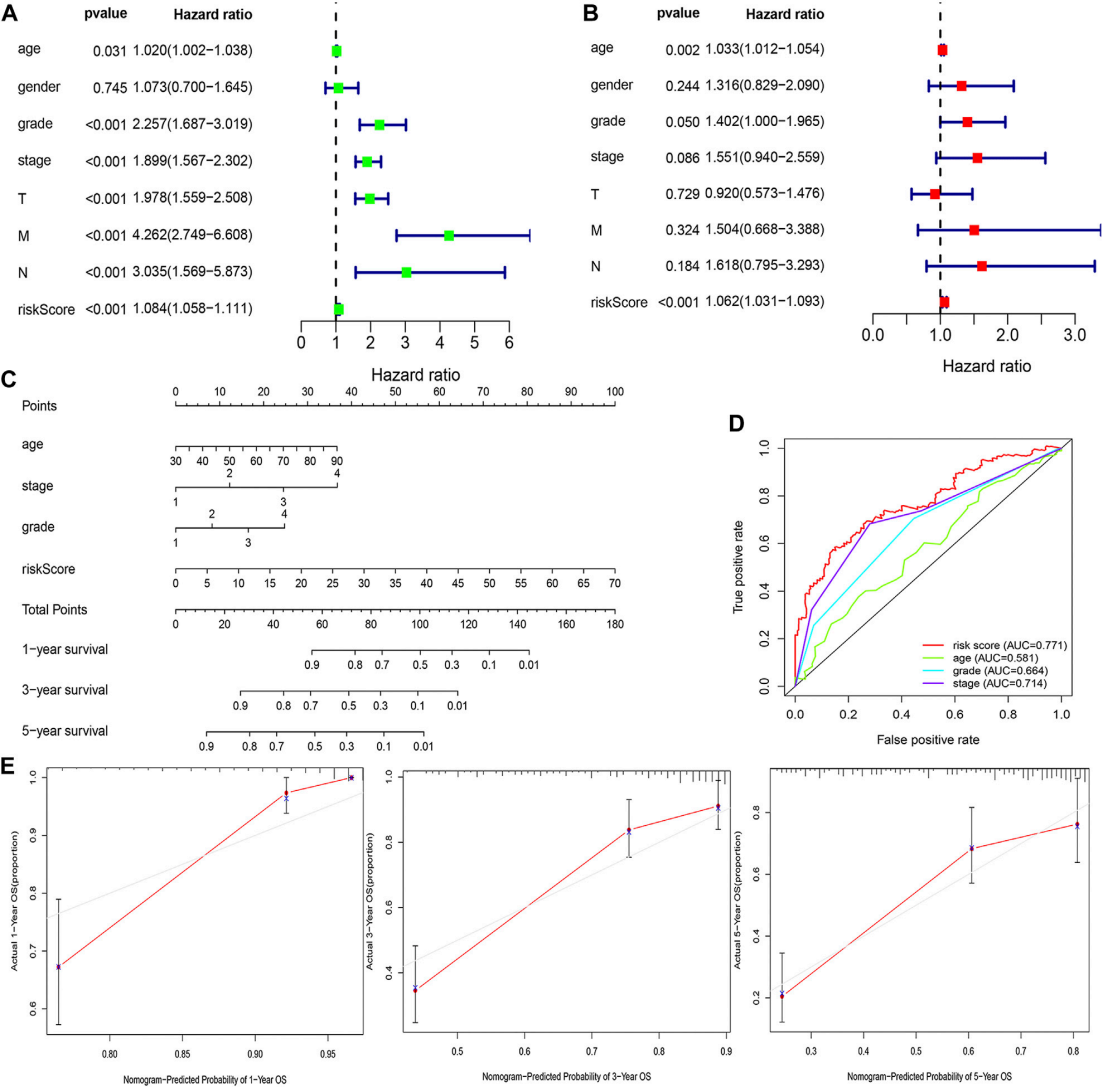
Estimation of the clinical value of the FRlncRNA signature in ccRCC patients. The univariate Cox regression analysis showed that risk score and clinicopathological features including gender, age, grade, stage, T stage, N stage, and M stage were prognostic-related variables **(A)**. The multivariate Cox regression analysis showed the risk score was an independent prognostic factor **(B)**. Construction of a prognostic nomogram based on the risk score and clinicopathological parameters to predict 1-, 3-, 5-year OS of ccRCC patients **(C)**. The multivariate ROC curve showed predictive accuracy of the risk score was similar to other clinicopathological features **(D)**. The calibration curves of the nomogram displayed the concordance between predicted and observed 1-, 3-, and 5-year OS **(E)**.

The risk score was demonstrated by the univariate and multivariate Cox regression analyses to be an independent prognostic factor (*p* < 0.05) (Supplementary Table S3, Figures 6A,B). Subsequently, clinicopathological characteristics including the age, grade and stage, and risk score were applied to construct the nomogram using the “rms” package in R software to predict the 1-, 3-, and 5-year OS of ccRCC patients (Figure 6C). The increasing risk score deteriorated the prognosis of ccRCC. The results of the multivariate ROC curve suggested that the AUC value was 0.771, which was higher than that of the grade (0.664) and stage (0.714), implying that the nomogram had the ability of accurate prediction for survival outcomes of ccRCC (Figure 6D). We used the calibration curve to observe whether the actual prognostic value was consistent with the predicted value of the nomogram and found that the calibration curves of 1-, 3-, and 5-year survival rates were consistent with the nomogram (Figure 6E). The clinical influences of the risk score for ccRCC patients in the training, testing, and overall cohorts are showed in Supplementary Table S4.

### Functional Analysis of the FRlncRNA Signature and FRlncRNA-Related FRGs

**FIGURE 7:**
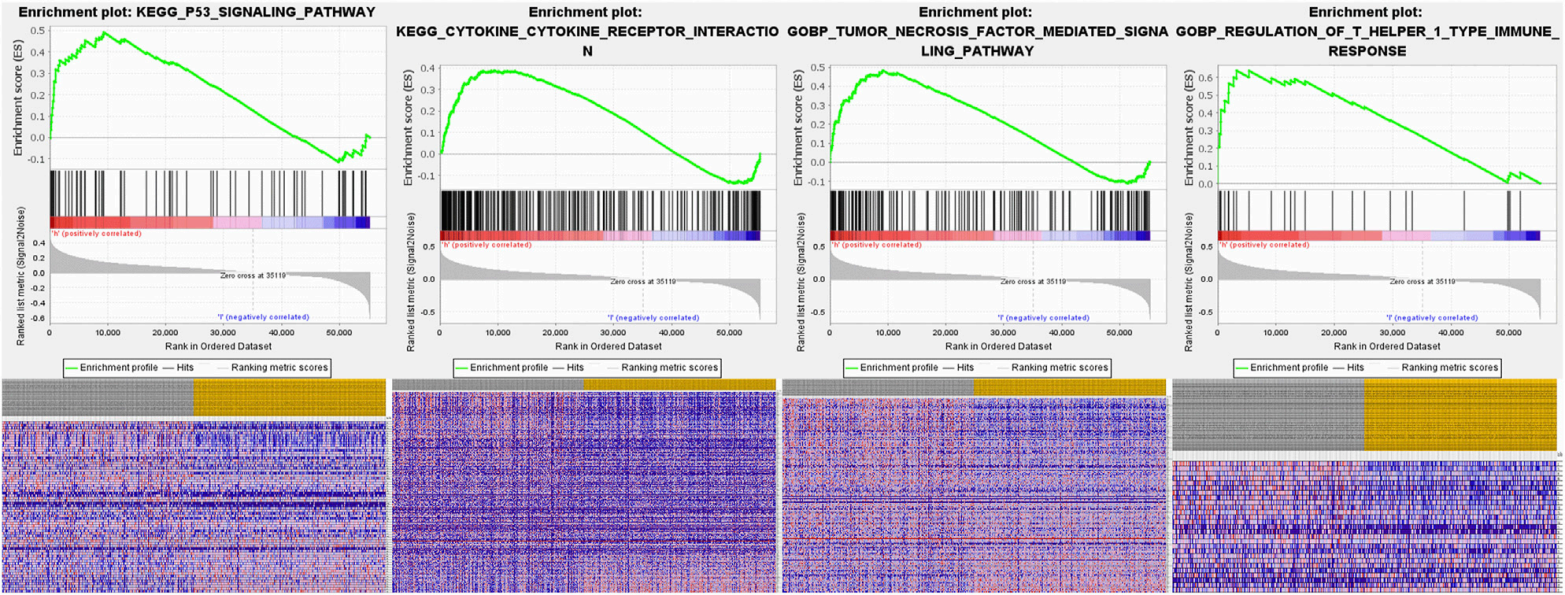
GSEA analysis of the high-risk group in ccRCC patients based on the prognostic signature.

The underlying molecular mechanisms of the FRlncRNA signature involved in the high-risk group were further verified by GSEA . Signaling pathways including the P53 signaling pathway [enrichment score (ES) 0.49; normalized enrichment score (NES) 1.76; nominal (NOM) *p*-value 0.03], the cytokine–cytokine receptor interaction signaling pathway (ES 0.39; NES 1.71; NOM *p*-value 0.03), the tumor necrosis factor–mediated signaling pathway (ES 0.50; NES 1.87; NOM *p*-value 0.005), and regulation of the T helper 1 type immune response signaling pathway (ES 0.64; NES 1.98; NOM *p*-value 0.007) were markedly enriched in the high-risk group (Figure 7).

**FIGURE 8:**
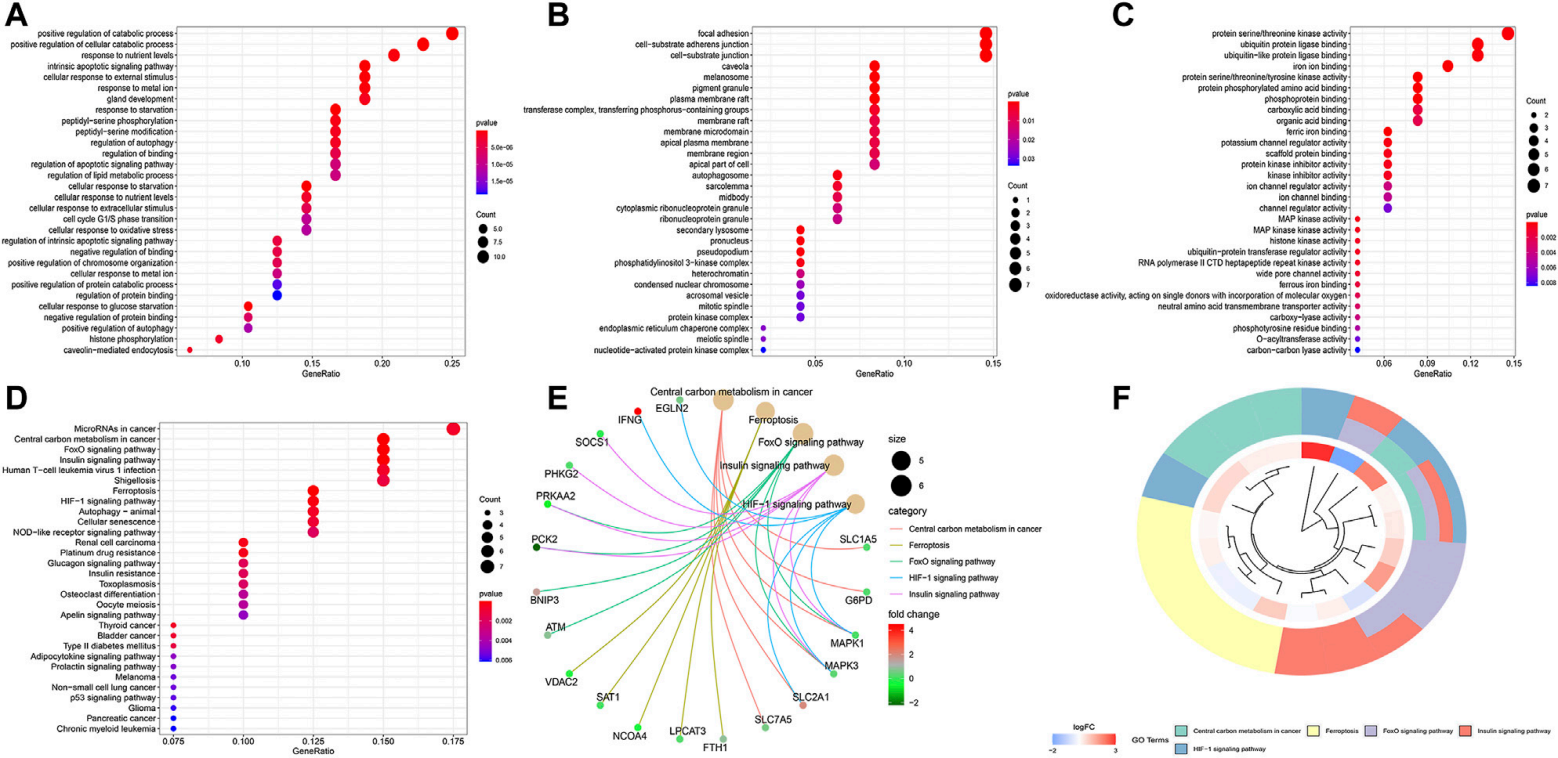
Functional enrichment analysis of FRG-associated eight FRlncRNAs. **(A)** biological process, **(B)** cellular component, **(C)** molecular function, **(D)** dotplot of the KEGG signal pathway showing the counts of genes, **(E)** Cnetplot of the KEGG signal pathway showing the “pathway-gene” network, and **(F)** Circos plot of the KEGG pathway enrichment results.

The top 30 terms from the GO analysis of FRlncRNA-related FRGs are demonstrated in the dot plot (Figures 8A–C). GO analysis consisted of biological process (BP) analysis mainly including positive regulation of the catabolic process and intrinsic apoptotic signaling pathway; cellular component (CC) analysis mainly containing focal adhesion, cell-substrate adherens junction, and cell-substrate junction; and molecular function (MF) analysis mostly composing of protein serine/threonine kinase activity, ubiquitin protein ligase binding, and iron ion binding. The “pathway-gene network” and “pathway-gene clustering,” as shown in Figures 8D–F, were plotted to represent the complex relationship between FRlncRNA-related FRGs and KEGG pathways.

### Construction of the Co-Expression Network and Verification of Major Genes

**FIGURE 9:**
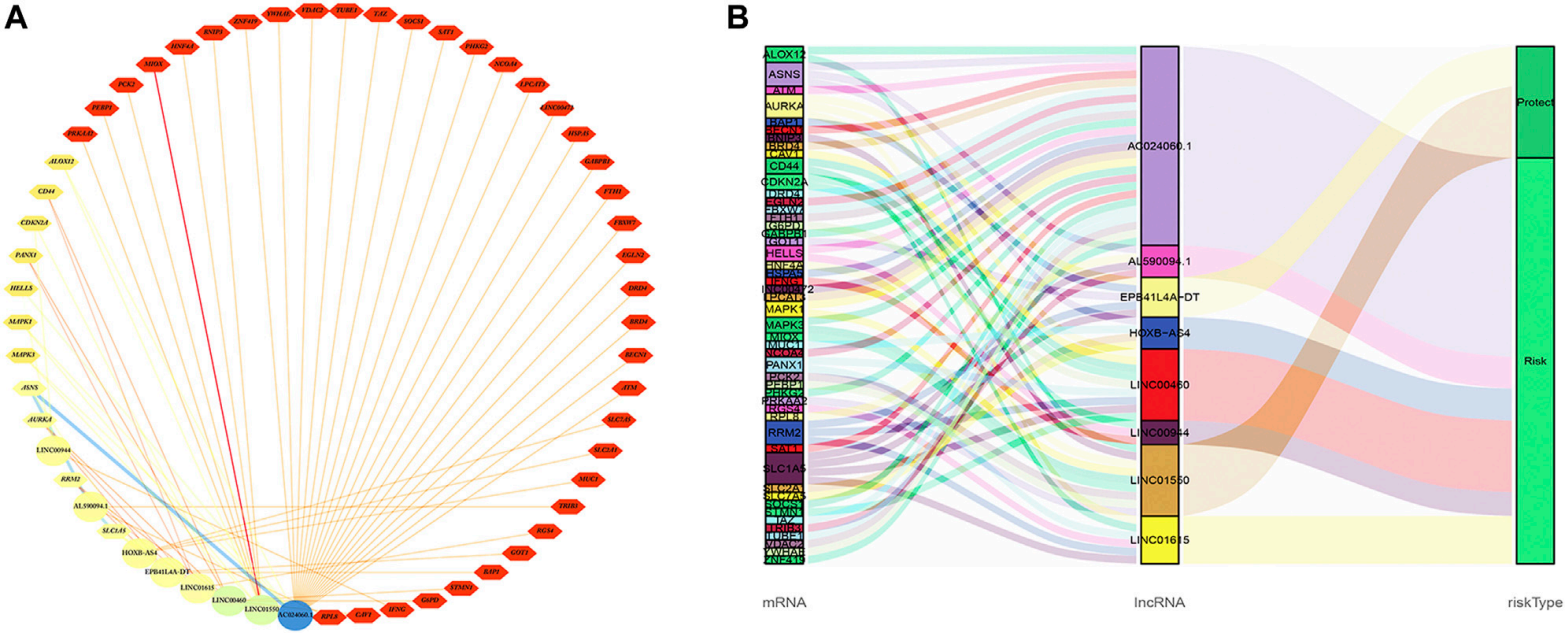
Co-expression network of eight FRlncRNAs and 49 FRGs **(A)**. The Sankey diagram showed the connective degree between 49 FRGs and eight FRlncRNAs (risk/protective) **(B)**.

The co-expression network between eight FRlncRNAs and 49 FRGs (*R* ^2^ > 0.3 and *p* < 0.001) is shown in Figure 9A. The Sankey diagram showed the interrelation between eight FRlncRNAs, 49 FRGs, and the risk type (Figure 9B). By analyzing the results of differential expression analysis (*p* < 0.01) and searching relevant literatures (Shijie Zhang et al., 2021; Chen and Zheng, 2021; Xuan et al., 2021) and databases, we selected four FRlncRNAs (LINC00460, LINC00944, LINC01550, and EPB41L4A-DT) for further study. Compared with normal tissues, LINC00460 (logFC = 5.039, *p* = 1.74E-19) and LINC00944 (logFC = 3.906, *p* = 2.46E-32) were significantly upregulated in ccRCC tissues, and LINC01550 (logFC = 0.359, *p* = 0.005) and EPB41L4A-DT (logFC = 0.2400, *p* = 0.003) were significantly downregulated in ccRCC tissues. To more accurately predict their action pathways, the ceRNA network (lncRNA–miRNA–mRNA) of four FRlncRNAs was explored (Supplementary Figure S3).

**FIGURE 10:**
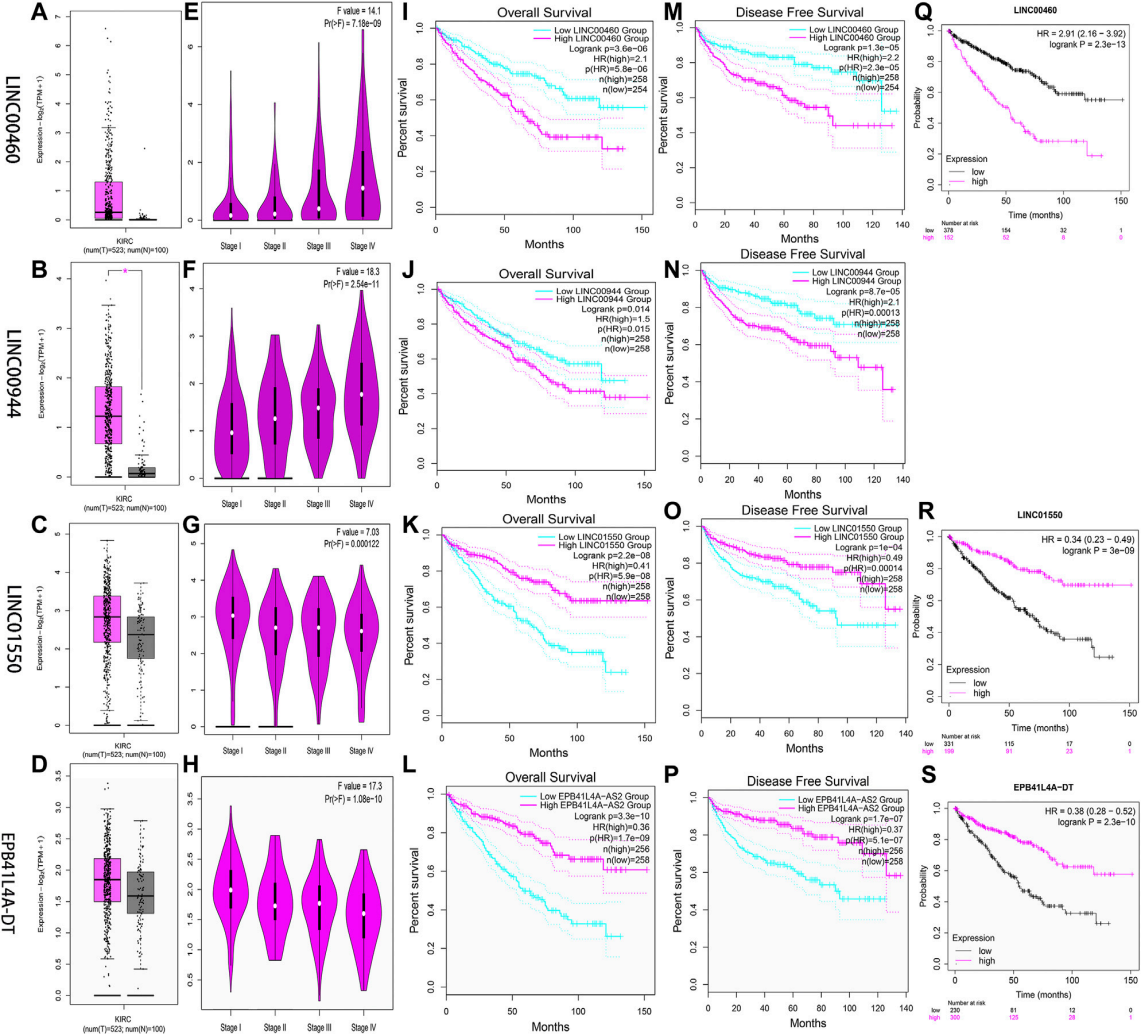
Verification of expression and prognosis of four FRlncRNAs (LINC00460, LINC00944, LINC01550, and EPB41L4A-DT) from the GEPIA **(A-P)** and K-M Plotter databases **(Q-S)**. The expression levels of LINC00460 and LINC00944 increased gradually with the increase of stages, but LINC01550 and EPB41L4A-DT decreased gradually with the increase of stages. The high expression levels of LINC00460 and LINC00944 and low expression levels of LINC01550 and EPB41L4A-DT were associated with poor prognosis (*p* < 0.05).

Two databases (GEPIA and K-M Plotter) were employed to investigate four FRlncRNAs including expression levels and survival outcomes. The expression levels of LINC00460 (Pr = 7.18E-09) and LINC00944 (Pr = 2.54E-11) increased gradually with the increase of stages but those of LINC01550 (Pr = 0.000122) and EPB41L4A-DT (Pr = 1.06E-10) decreased gradually with the increase in stages, suggesting that four FRlncRNAs were strongly correlated with tumor progression (Figures 10A–H). High expression levels of LINC00460 and LINC00944 and low expression levels of LINC01550 and EPB41L4A-DT were related to the poor prognosis (*p* < 0.05) (Figures 10I–P). Similarly, lower 5-year OS (*p* < 0.05) was noted in 530 ccRCC patients from the K-M Plotter database with an increasing expression of LINC00460 or decreasing expressions of LINC01550 and EPB41L4A-DT (Figures 10Q–S).

**FIGURE 11:**
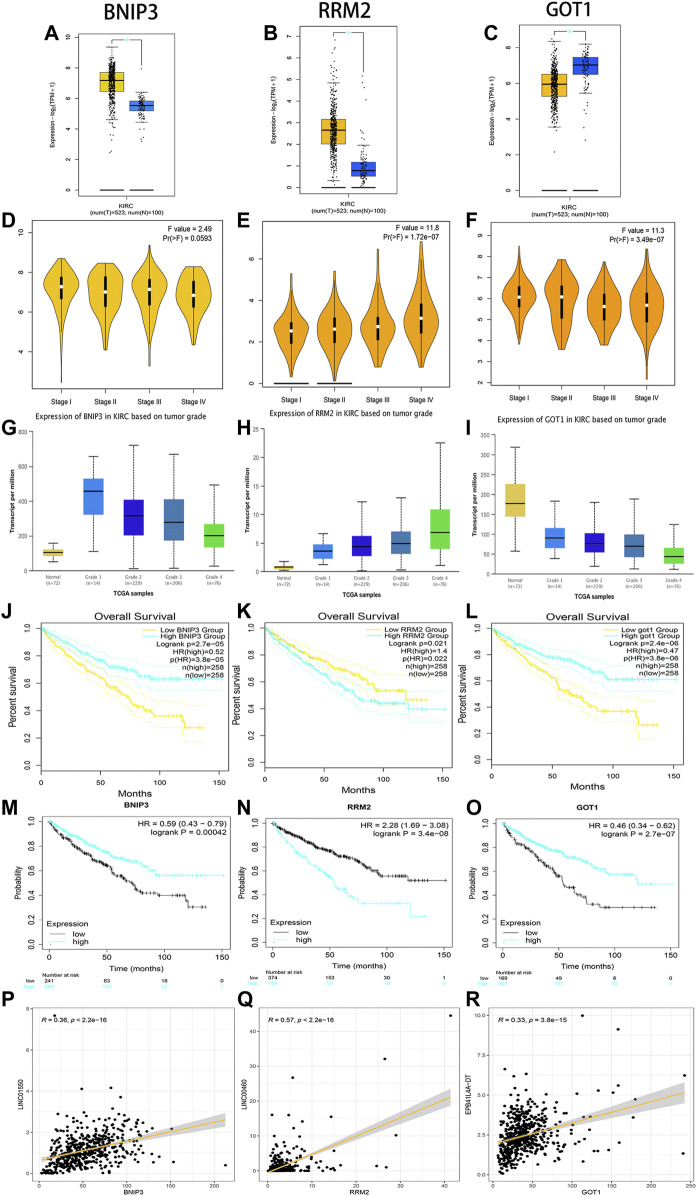
Verification of expression and prognosis of three target genes (BNIP3, RRM2, and GOT1) from the GEPIA, UALCAN, and K-M Plotter databases. BNIP3 and RRM2 were significantly upregulated in ccRCC tissues, but GOT1 was significantly downregulated in ccRCC tissues **(A-C)**. The expression levels of BNIP3 and GOT1 decreased gradually with the increase of grades, but RRM2 increased gradually with the increase of grades and stages **(D-I)**. The high expression levels of RRM2 and low expression levels of BNIP3 and GOT1 were associated with poor prognosis **(J-O)**. Linear correlation analysis found that RRM2, BNIP3, and GOT1 may be potential target genes of LINC00460, LINC01550, and EPB41L4A-DT, respectively **(P-R)**.

Through linear correlation analysis (R > 0.3 and *p* < 0.001) and literature retrieval (Shao et al., 2019; Xiong et al., 2021a; Hong et al., 2021), we selected three target genes (BNIP3, RRM2, and GOT1) for further verification. BNIP3 (*p* < 0.05) and RRM2 (*p* < 0.05) were significantly upregulated in ccRCC tissues, but GOT1 (*p* < 0.05) was significantly downregulated in ccRCC tissues (Figures 11A–C). The expression levels of BNIP3 and GOT1 decreased gradually with the increase in grades, but RRM2 increased gradually with the increase in clinical grades and stages, which revealed that three target genes were closely related to tumor progression (Figures 11D–I). High expression levels of RRM2 and low expression levels of BNIP3 and GOT1 were related to poor prognosis after the analysis of GEPIA and K-M Plotter databases (*p* < 0.05) (Figures 11J–O). The linear correlation analysis found that RRM2, BNIP3, and GOT1 may be potential targets of LINC00460 (R = 0.35; *p* < 2.2E-16), LINC01550 (R = 0.57; *p* < 2.2E-16), and EPB41L4A-DT (R = 0.33; *p* < 3.8E-15), respectively, which also needed further experimental verification (Figures 11P–R).

### Tumor Tissue Validation

**FIGURE 12:**
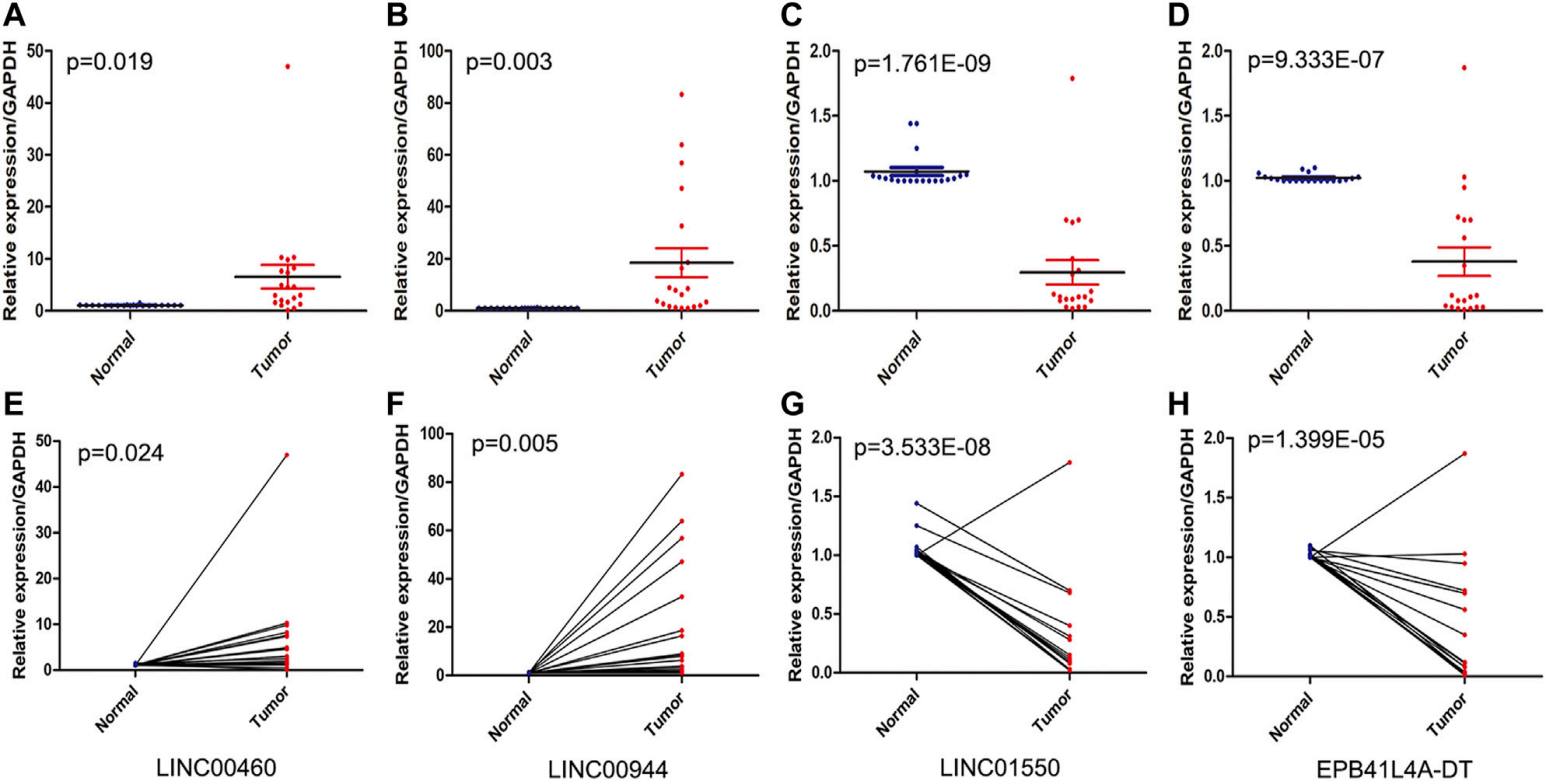
QRT-PCR analysis of the expression of four FRlncRNAs (LINC00460 **(A, E)**, LINC00944 **(B, F)**, LINC01550 **(C, G)**, and EPB41L4A-DT **(D,H)**) from twenty patients with ccRCC.

The expression levels of four FRlncRNAs were verified by qRT-PCR in the tumor and adjacent normal tissues collected from twenty ccRCC patients (Supplementary Table S5). LINC01550 and EPB41L4A-DT in tumor tissues were downregulated, while LINC00460 and LINC00944 were upregulated, showing the statistical significance (Figures 12A–D, t-test, *p* < 0.05; Figures 12E–H, paired t-test, *p* < 0.05). These results were consistent with our previous verification results, but more samples were still needed for verification.

### Immune Analysis

**FIGURE 13:**
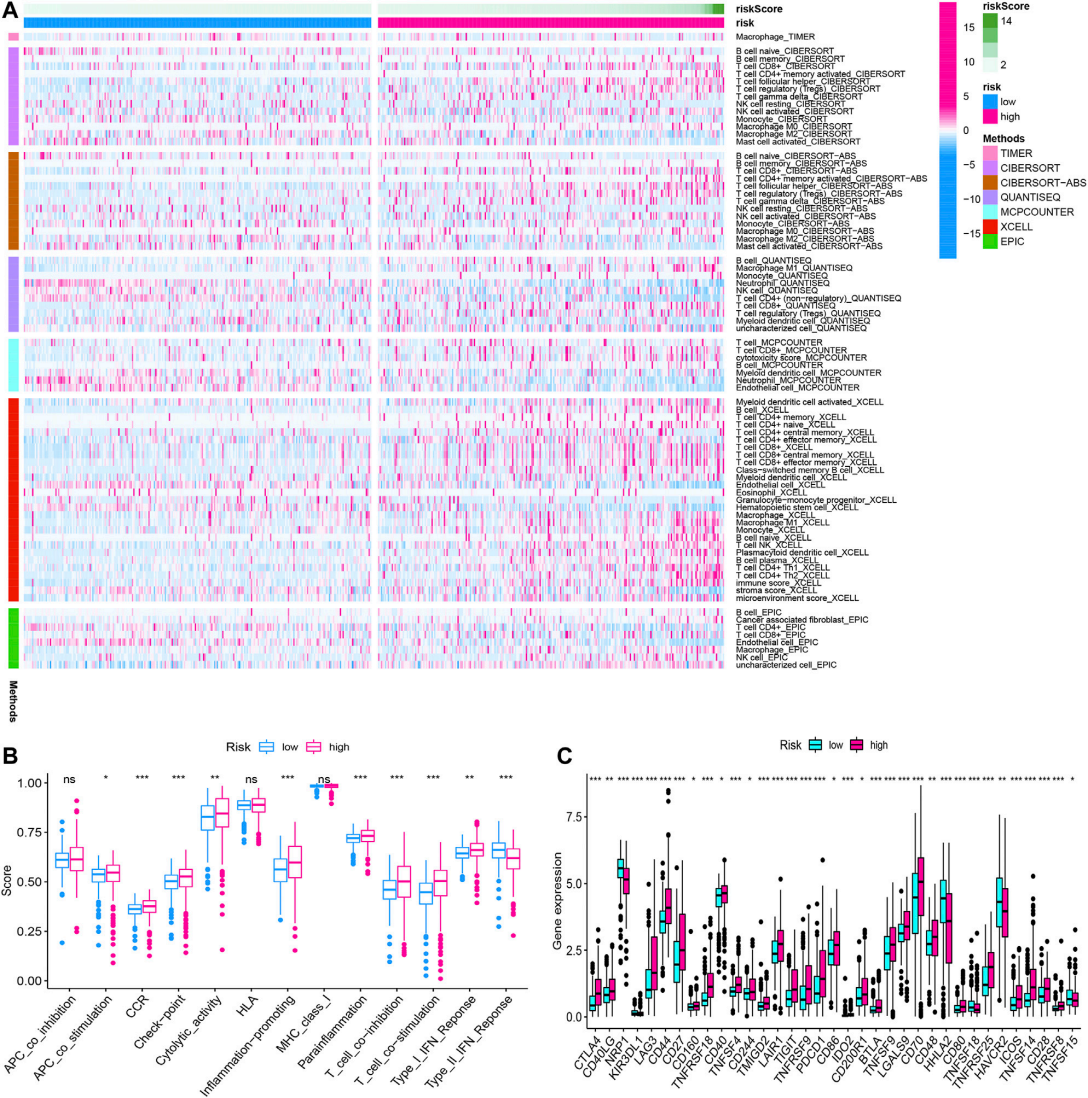
The immune infiltration, **(A)** immune function, **(B)** immune checkpoint, and **(C)** of the high- and low-risk groups for BLCA patients in the overall cohorts. *p < 0.05; **p < 0.01; ***p < 0.001.

We summarized the result of 507 ccRCC patients calculated by various algorithms and compared all the immune cell subtypes in two groups. Infiltration proportion of partial cell subtypes had an obvious difference between two groups, among which mainly T cell NK, B cell, T cell follicular helper, and T cell regulatory (Tregs) had a higher infiltration proportion in the high-risk group, while T cell CD4^+^, neutrophils and endothelial cells had a lower proportion (Figure 13A). The immune functions of the high-risk group and low-risk group were analyzed, respectively, using “GSVA” and “GSEABase” packages in R software and found that almost all items (APC co-stimulation, CCR, check-point, cytolytic activity, inflammation promoting, para inflammation, T-cell co-inhibition, T-cell co-stimulation, Type I IFN response) in the high-risk group were upregulated (*p* < 0.05, Figure 13B), indicating a significant change in the immunophenotype in the high-risk group. We further explored the expression of immune checkpoint-related markers in two groups and found some markers (CTLA4, CD40LG, LAG3, CD44, CD27, CD160, TNFRSF18, CD40, TNFSF4, CD244, TMIGD2, LAIR1, TIGIT, TNFRSF9, PDCD1, CD86, IDO2, CD200R1, BTLA4, TNFSF9, LGALS9, CD70, CD48, CD80, TNFRSF25, ICOS, TNFSF14, CD28, and TNFRSF8) in the high-risk group were upregulated, and some markers (NRP1, KIR3DL1, HHLA2, TNFSF18, HAVCR2, and TNFSF15) were downregulated, indicating an immunosuppressive and exhausted phenotype in the high-risk group (Figure 13C). Based on the above analyses, we found two groups had a significant distinct pattern of immune infiltration, which may lead to different survival benefits.

## Discussion

Although breakthroughs have been made in surgical methods and postoperative auxiliary regimens for ccRCC in recent years, the prognosis of patients with advanced ccRCC and metastatic ccRCC was still of concern (Atkins and Tannir, 2018; Toth and Cho, 2020). Furthermore, some ccRCC patients with the same TNM stage or similar risk factors may represent different clinical outcomes due to complicated pathogenic molecules. It was necessary to discover molecular biomarkers that can predict the prognosis of the tumor (Attalla et al., 2020). Ferroptosis was reported to be closely associated with the biological process of ccRCC, for example proliferation, invasion, and metastasis (Miess et al., 2018; Markowitsch et al., 2020; Tang and Xiao, 2020), in which crucial regulatory roles of lncRNAs were identified in the ferroptosis-related biological process of malignant tumor cells (Mao et al., 2018; Wang et al., 2019; Wu and Liu, 2021). So an FRlncRNA signature was established and evaluated in predicting clinical outcomes.

Fifteen FRlncRNAs related with prognosis of ccRCC were initially identified in the training group, and a prognostic signature was constructed containing eight FRlncRNAs using multivariate Cox regression along with the Lasso regression. The OS of patients with high-risk scores was shorter than that of patients with low-risk scores. The FRlncRNA signature validated by the ROC curve was greatly sensitive and act as specific prognosis markers for ccRCC, which further verifications were performed in the testing cohorts, overall cohorts, and ICGC cohorts. The FRlncRNA signature was also related to different OS in various subgroups of ccRCC, for instance age, gender, grade, stage, T stage, and M stage. Importantly, this signature was proved to be an independent risk factor in predicting survival outcomes. Next, we set up a nomogram including the risk score to calculate 1-, 3-, and 5-year survival rates of ccRCC patients, which had a higher sensitivity compared with the conventional grade and stage standard. After co-expression network construction and differential expression analysis, we selected four FRlncRNAs (LINC00460, LINC00944, LINC01550, and EPB41L4A-DT) which were closely related with FRGs for further study. The expression levels of four FRlncRNAs were verified by qRT-PCR from twenty ccRCC patients, which found that LINC01550 and EPB41L4A-DT in tumor tissues were downregulated, while LINC00460 and LINC00944 were upregulated. The expression levels of LINC00460, LINC00944, LINC01550, and EPB41L4A-DT differed across the four stages, suggesting that four FRlncRNAs were closely related to the tumor stage. Two external databases confirmed that four FRlncRNAs were significantly correlated with the prognosis of ccRCC. The correlation analysis identified that RRM2, BNIP3, and GOT1 may be potential targets of LINC00460, LINC01550, and EPB41L4A-DT, respectively. BNIP3 and RRM2 strikingly increased in ccRCC tissues, while GOT1 significantly reduced. BNIP3 and GOT1 were downregulated gradually with the increase in the grade, but RRM2 increased gradually with the increase in the grade and stage, which revealed that three target genes were closely related with tumor progression. The high expression level of RRM2 and low expression levels of BNIP3/GOT1 were related to poor prognosis, which was consistent with the prognosis of three FRlncRNAs after the analysis of GEPIA and K-M Plotter databases.

Based on the functional annotation and pathway enrichment analysis of the FRlncRNA signature, the mechanism of FRlncRNAs regulating ccRCC development was intuitively outlined. The results suggest that FRlncRNAs can positively regulate the P53 signaling pathway, tumor necrosis factor (TNF)-mediated signaling pathway, cytokine–cytokine receptor interaction, and T helper 1 type immune response signaling pathway. ​In addition, the GSEA found FRlncRNA-related FRGs were significantly enriched on the microRNA in cancer, iron ion binding, ferroptosis, focal adhesion, positive regulation of the catabolic process, and hypoxia-inducible transcription factor 1 (HIF-1) signaling pathway. We further constructed the ceRNA network to reveal the potential pathway of four major FRlncRNAs through acting on microRNA. P53, as an important regulatory factor in the development of cancer, often played a role as a target protein in ccRCC (Huang et al., 2020; Patergnani et al., 2020; Chen et al., 2021a; Sekino et al., 2021a). P53 knockout decreased sensitivity to sunitinib, and p53-positive cases tended to be associated with poor progression-free survival after first-line sunitinib treatment (Sekino et al., 2021b). The TNF-family-related signature of ccRCC also was proved to be closely related to the prognostic value, immune infiltration, and tumor mutation burden (Wenhao Zhang et al., 2021).

Ferroptosis was first reported in non–small cell lung cancer cells (Dixon et al., 2012). Subsequently, researchers examined the sensitivity of 117 cancer cells to erastin-induced ferroptosis cell death and found that RCC was particularly sensitive to GPX4-regulated ferroptosis (Yang et al., 2014). Miess et al. reported that the induction of silencing of glutathione peroxidase, GPx3, and GPx4 genes by siRNA was lethal to renal cancer cells (Miess et al., 2018). The impaired fatty acid degradation might drive ccRCC cells to become extremely dependent on GSH synthesis to prevent the accumulation of lipid peroxides and maintain cell viability owing to HIF-induced lipid uptake. These findings suggested that ccRCC cells were sensitive to ferroptosis-mediated cell death by inhibiting or even blocking GSH synthesis of tumor cells (Tang and Xiao, 2020). Notably, RCC cells re-expressing Von Hippel-Lindau developed resistance to ferroptosis. Yang et al. reported that TAZ, an effector of the Hippo pathway, regulated the sensitivity of RCC cells to ferroptosis (Yang et al., 2019). Therefore, modulating ferroptosis may have therapeutic potentials.

Currently, four FRlncRNAs (LINC01615, LINC01550, EPB41L4A-DT, and LINC00944) have been reported to be related to cancer development. Among them, LINC01615 was not only an optimal diagnostic lncRNA biomarker for head and neck squamous cell carcinoma but also closely related to survival time (Hu et al., 2020). LINC01615 has also been identified to be associated with the extracellular matrix and had further impacts on the metastasis of hepatocellular carcinoma (Ji et al., 2019). In addition, Chen et al. identified that the increased expression of LINC01550 seriously impeded cell proliferation and invasion abilities and caused cell apoptosis and G1 and S-phase arrest of the melanoma cells, which indicated that LINC01550 may act as a potential therapeutic target for melanoma (Chen et al., 2021b). Importantly, the overexpressed lncRNA EPB41L4A-DT in the renal cancer cell line 786-O, cell proliferation assays, flow cytometry, and clonogenic assay showed that upregulating EPB41L4A-DT may inhibit the proliferation of renal cancer cells (Xu et al., 2016). The knockdown of LINC00944 in 786-O and 769-P RCC cells could significantly decrease proliferation and migration and also promoted phosphorylation of Akt (Chen and Zheng, 2021). The above results further confirmed the accuracy of our model, but the potential mechanism of LINC01615 and LINC01550 in the occurrence and development of RCC needed to be further studied.

Besides, two FRlncRNAs (AC024060.1 and LINC00460) have been included in related clinical prediction models and demonstrated a good predictive power for the prognosis of cancer patients. Among them, AC024060.1 may predict the prognosis and progression of patients with bladder cancer as an immune-related lncRNA (Wang et al., 2021). Also, AC024060.1 as an autophagy-related lncRNA may be involved in the diagnosis and prognosis of bladder cancer (Wan et al., 2021). Moreover, Zhang et al. constructed a prognostic model based on ceRNA-related lncRNA and found that LINC00460 may provide insight into the prognostic biomarkers and therapeutic targets of ccRCC (Zhang et al., 2020). Little is known about prognostic effects of AL590094.1 and HOXB-AS4 on the prognosis of cancer. Therefore, more research is needed to explore the influence of FRlncRNAs on the prognosis of ccRCC mediated by ferroptosis.

There are some basic experimental studies on the biological function of two target genes (RRM2 and BNIP3) regulated by FRlncRNAs for renal cancer. Xiong et al. reported that RRM2 could regulate the sensitivity of renal cancer to sunitinib and PD-1 blockade *via* the stabilization of ANXA1 and the activation of the AKT pathway, and the effectiveness of the PD-1 blockade was improved by the deletion of RRM2 (Xiong et al., 2021b). Shao et al. identified that BNIP3 inactivation in renal cancer was probably caused by histone deacetylation, rather than methylation, and the histone deacetylation inhibitor can restore the expression of BNIP3 in renal cancer, subsequently resulting in growth inhibition and apoptotic promotion (Shao et al., 2019). These findings were consistent with our results, which may also need further experimental verification. As FRGs, GOT1 has only been proved to be a biomarker of ccRCC patients in clinical prediction models, which also needs further basic experiments to explore (Chang et al., 2021; Hong et al., 2021).

Ferroptosis is a type of cell death providing novel insights into tumor therapy. However, there are still many crucial questions less well studied, such as the interaction between ferroptosis and other cell deaths and the host immunogenicity. Thus, this study examined ferroptosis biomarkers can serve as a predictive factor to ccRCC prognosis, which may offer consultations for therapeutic modalities. Nevertheless, the current study still has some shortcomings. First, due to the shortage of the single and small amount data source, a certain deviation may occur in this study. Second, to illustrate the prognostic function of ferroptosis-related signals, more prospectives are required to cooperate with our retrospective study. Third, with the in-depth study of FRGs, the study needs to be updated regularly. Fourth, relevant functional assays should be conducted to examine the ways that FRlncRNAs influence the development and progression of ccRCC and explore underlying molecular mechanisms. Fifth, this model divided patients into the high-risk group and low-risk group according to the median risk score, although better sensitivity and specificity were obtained; the accuracy was worse than the optimal cutoff value.

## Conclusion

The FRlncRNA signature was accurate and act as reliable tools for predicting clinical outcomes and the immune microenvironment of patients with ccRCC, which may be molecular biomarkers and therapeutic targets.

## Data Availability

The original contributions presented in the study are included in the article/Supplementary Material, further inquiries can be directed to the corresponding author.
