# Supplementary material for: Identification and Validation of a Ferroptosis-Related Long Non-Coding RNA (FRlncRNA) Signature to Predict Survival Outcomes and the Immune Microenvironment in Patients With Clear Cell Renal Cell Carcinoma
Source: Front Genet. 2022 Mar 8;13:787884. doi: 10.3389/fgene.2022.787884 (PMC8957844; doi:10.3389/fgene.2022.787884)
Supplement: Supplementary file 1 [file DataSheet3.ZIP › Supplementary Table/Supplementary Table 1.docx]

Supplementary **Table 1. The HRs, P-values, and Coefficients of 8 ferroptosis-related lncRNAs.**

| **lncRNA** | **Coefficient** | **HR** | **95% CI of HR** | **p-value** |
| --- | --- | --- | --- | --- |
| **AL590094.1** | 0.190 | 1.210 | 1.013-1.445 | 0.036 |
| **LINC00460** | 0.047 | 1.048 | 1.003-1.095 | 0.035 |
| **LINC00944** | 0.204 | 1.226 | 1.069-1.407 | 0.004 |
| **AC024060.1** | 0.168 | 1.183 | 1.110-1.261 | <0.001 |
| **EPB41L4A-DT** | -0.134 | 0.875 | 0.726-1.054 | 0.159 |
| **HOXB-AS4** | 0.116 | 1.123 | 1.009-1.249 | 0.033 |
| **LINC01550** | -0.345 | 0.708 | 0.519-0.965 | 0.029 |
| **LINC01615** | 0.048 | 1.050 | 1.006-1.095 | 0.024 |

**HR, hazard rate; CI, confidence interval;**
