# Supplementary material for: Identification and Validation of a Ferroptosis-Related Long Non-Coding RNA (FRlncRNA) Signature to Predict Survival Outcomes and the Immune Microenvironment in Patients With Clear Cell Renal Cell Carcinoma
Source: Front Genet. 2022 Mar 8;13:787884. doi: 10.3389/fgene.2022.787884 (PMC8957844; doi:10.3389/fgene.2022.787884)
Supplement: Supplementary file 1 [file DataSheet3.ZIP › Supplementary Table/Supplementary Table 2.docx]

**Supplementary Table 2. The comparison of studies about existing ferroptosis-related signatures for ccRCC.**

| References | Signature | Database | Gene list | Survival event | AUC value |
| --- | --- | --- | --- | --- | --- |
| Xing et al (2021) | Ferroptosis-related lncRNA | TCGA | DUXAP8, LINC02609, LUCAT1 | OS | 0.7196 |
| Ma et al (2021) | Ferroptosis-related gene | TCGA | AKR1C1, CARS1, CD44, DPP4, FANCD2, HMGCR, MT1G, NCOA4, SLC7A11, ACACA, NOX1, GOT1 | OS | 0.782 |
| Hong et al (2021) | Ferroptosis-related gene | TCGA | CARS1, HMGCR, CHAC1, GOT1, CD44, STEAP3, AKR1C1, CBS, DPP4, FANCD2, SLC1A5, NCOA4 | OS | 0.761 |
| Zheng et al (2021) | Ferroptosis-related gene | TCGA | CD44, DPP4, NCOA4, SLC7A11 | OS | 0.756 |
| Li et al (2021) | Ferroptosis-related gene | TCGA | HMGCR, MT1G, BID, EIF4A1, FOXM1, TFAP2C, CHAC1 | OS | 0.773 |
| Yu et al (2021) | Feature genes related to ccRCC | TCGA  GEO | CTLA-4, BIRC5, HLA-G, DLGAP5, PLK1, CD44, MAD2L1, HLA-DRA | OS | 0.77 |
| Our study | Ferroptosis-related lncRNA | TCGA  ICGC | AL590094.1, LINC00460, LINC00944, AC024060.1, HOXB-AS4, LINC01615, EPB41L4A-DT, LINC01550 | OS | 0.875 |

LncRNA, long non-coding RNA; TCGA, The Cancer Genome Atlas; AUC, area under the curve; OS, overall survival; ccRCC, clear cell renal cell carcinoma.
