# Supplementary material for: Identification and Validation of a Ferroptosis-Related Long Non-Coding RNA (FRlncRNA) Signature to Predict Survival Outcomes and the Immune Microenvironment in Patients With Clear Cell Renal Cell Carcinoma
Source: Front Genet. 2022 Mar 8;13:787884. doi: 10.3389/fgene.2022.787884 (PMC8957844; doi:10.3389/fgene.2022.787884)
Supplement: Supplementary file 1 [file DataSheet3.ZIP › Supplementary Table/Supplementary Table 3.docx]

**Supplementary Table 3. Univariate and multivariate Cox regression analysis of the association between clinicopathological factors (including risk score) and OS of ccRCC patients in the training, testing and overall cohorts.**

| **Variable** | **Overall cohorts** | | | | **Training cohorts** | | | | **Testing cohorts** | | | |
| --- | --- | --- | --- | --- | --- | --- | --- | --- | --- | --- | --- | --- |
|  | **Univariate** | | **Multivariate** | | **Univariate** | | **Multivariate** | | **Univariate** | | **Multivariate** | |
|  | **HR**  **(95% CI)** | **P** | **HR**  **(95% CI)** | **P** | **HR**  **(95% CI)** | **P** | **HR**  **(95% CI)** | **P** | **HR**  **(95% CI)** | **P** | **HR**  **(95% CI)** | **P** |
| **Age** | 1.020 (1.002-1.038) | 0.031 | 1.033 (1.012-1.054) | 0.002 | 1.028 (1.001-1.055) | 0.039 | 1.059 (1.025-1.094) | <0.001 | 1.013 (0.989-1.037) | 0.305 | 1.019 (0.992-1.047) | 0.162 |
| **Gender** | 1.073 (0.700-1.645) | 0.745 | 1.316 (0.829-2.090) | 0.244 | 1.016 (0.538-1.918) | 0.960 | 1.222 (0.569-2.624) | 0.607 | 1.106 (0.620-1.972) | 0.734 | 0.979 (0.520-1.840) | 0.947 |
| **Grade** | 2.257 (1.687-3.019) | <0.001 | 1.402 (1.000-1.965) | 0.050 | 2.046 (1.330-3.148) | 0.001 | 1.367 (0.817-2.288) | 0.233 | 2.453 (1.647-3.652) | <0.001 | 1.535 (0.904-2.606) | 0.113 |
| **AJCC stage** | 1.899 (1.567-2.302) | <0.001 | 1.551 (0.940-2.559) | 0.086 | 2.078 (1.511-2.858) | <0.001 | 0.709 (0.262-1.913) | 0.497 | 1.881 (1.444-2.451) | <0.001 | 2.225 (0.946-5.236) | 0.067 |
| **T stage** | 1.978 (1.559-2.508) | <0.001 | 0.920 (0.573-1.476) | 0.729 | 2.774 (1.753-4.388) | <0.001 | 2.646 (1.144-6.119) | 0.023 | 1.702 (1.267-2.286) | <0.001 | 0.531 (0.227-1.241) | 0.144 |
| **N stage** | 3.035 (1.569-5.873) | <0.001 | 1.618 (0.795-3.293) | 0.184 | 3.456 (1.422-8.399) | 0.006 | 6.592 (2.088-20.807) | 0.001 | 2.300 (0.826-6.407) | 0.111 | 0.929 (0.269-3.213) | 0.908 |
| **M stage** | 4.262 (2.749-6.608) | <0.001 | 1.504 (0.668-3.388) | 0.324 | 3.340 (1.765-6.319) | <0.001 | 6.109 (1.272-29.346) | 0.024 | 6.440 (3.396-12.213) | <0.001 | 1.364 (0.357-5.208) | 0.650 |
| **Risk score** | 1.084 (1.058-1.111) | <0.001 | 1.062 (1.031-1.093) | <0.001 | 1.072 (1.039-1.105) | <0.001 | 1.045 (1.008-1.084) | 0.017 | 1.175 (1.101-1.254) | <0.001 | 1.088 (1.007-1.174) | 0.032 |

**TCGA, The Cancer Genome Atlas; HR, Hazard Ratio; AJCC, American Joint Committee on Cancer; CI, Confidence Intervals.**
