# Supplementary material for: Identification and Validation of a Ferroptosis-Related Long Non-Coding RNA (FRlncRNA) Signature to Predict Survival Outcomes and the Immune Microenvironment in Patients With Clear Cell Renal Cell Carcinoma
Source: Front Genet. 2022 Mar 8;13:787884. doi: 10.3389/fgene.2022.787884 (PMC8957844; doi:10.3389/fgene.2022.787884)
Supplement: Supplementary file 1 [file DataSheet3.ZIP › Supplementary Table/Supplementary Table 4.docx]

**Supplementary Table 4. Clinical influences of risk score for ccRCC patients in the training, testing and overall cohorts.**

| **Variable** | | **Risk score** | | | | | | | | | | | | |
| --- | --- | --- | --- | --- | --- | --- | --- | --- | --- | --- | --- | --- | --- | --- |
|  |  | **Overall cohorts** | | | | **Training cohorts** | | | | **Testing cohorts** | | | | |
|  |  | **n** | **Mean ± SD** | **t** | **p** | **n** | **Mean**  **± SD** | **t** | **p** | **n** | **Mean**  **± SD** | **t** | **p** |  |
| **Age** | **≤65** | 146 | 1.873 ± 5.553 | 0.007 | 0.995 | 62 | 2.586 ± 8.276 | -0.366 | 0.715 | 84 | 1.346 ± 1.693 | 0.652 | 0.517 |  |
|  | **>65** | 86 | 1.876 ± 3.133 |  |  | 34 | 2.170 ± 2.496 |  |  | 52 | 1.684 ± 3.497 |  |  |  |
| **Gender** | **Female** | 88 | 1.634 ± 2.911 | -0.687 | 0.493 | 34 | 1.799 ± 1.819 | -0.896 | 0.373 | 54 | 1.529 ± 3.438 | 0.178 | 0.859 |  |
|  | **Male** | 144 | 2.021 ± 5.647 |  |  | 62 | 2.790 ± 8.354 |  |  | 82 | 1.440 ± 1.716 |  |  |  |
| **Grade** | **I-II** | 101 | 0.968 ± 0.739 | -2.906 | 0.004 | 38 | 1.250 ± 0.890 | -0.717 | 0.091 | 63 | 0.797 ± 0.575 | -3.200 | 0.002 |  |
|  | **III-IV** | 131 | 2.573 ± 6.266 |  |  | 58 | 3.218 ± 8.655 |  |  | 73 | 2.060 ± 3.315 |  |  |  |
| **Stage** | **I-II** | 124 | 0.910 ± 0.601 | -3.135 | 0.002 | 42 | 1.012 ± 0.607 | -2.083 | 0.042 | 82 | 0.857 ± 0.595 | -3.002 | 0.004 |  |
|  | **III-IV** | 108 | 2.981 ± 6.845 |  |  | 54 | 3.549 ± 8.924 |  |  | 54 | 2.414 ± 3.781 |  |  |  |
| **T stage** | **T1-2** | 136 | 1.036 ± 0.926 | -2.732 | 0.007 | 50 | 1.277 ± 1.256 | -1.695 | 0.097 | 86 | 0.895 ± 0.638 | -2.827 | 0.007 |  |
|  | **T3-4** | 96 | 3.062 ± 7.224 |  |  | 46 | 3.702 ± 9.626 |  |  | 50 | 2.473 ± 3.918 |  |  |  |
| **N stage** | **N0** | 218 | 1.779 ± 4.856 | -1.594 | 0.130 | 88 | 2.377 ± 7.063 | -0.641 | 0.529 | 130 | 1.374 ± 2.363 | -1.173 | 0.546 |  |
|  | **N1** | 14 | 2.153 ± 2.095 |  |  | 8 | 3.123 ± 2.508 |  |  | 6 | 3.677 ± 4.784 |  |  |  |
| **M stage** | **M0** | 191 | 1.220 ± 1.307 | -2.228 | 0.032 | 74 | 1.366 ± 1.018 | -1.601 | 0.124 | 82 | 1.959 ± 2.722 | -1.300 | 0.293 |  |
|  | **M1** | 41 | 4.923 ± 10.627 |  |  | 22 | 6.046 ± 13.698 |  |  | 3 | 5.792 ± 5.079 |  |  |  |

**SD, Standard Deviation.**
