# Supplementary material for: Identification and Validation of a Ferroptosis-Related Long Non-Coding RNA (FRlncRNA) Signature to Predict Survival Outcomes and the Immune Microenvironment in Patients With Clear Cell Renal Cell Carcinoma
Source: Front Genet. 2022 Mar 8;13:787884. doi: 10.3389/fgene.2022.787884 (PMC8957844; doi:10.3389/fgene.2022.787884)
Supplement: Supplementary file 1 [file DataSheet3.ZIP › Supplementary Table/Supplementary Table 5.docx]

| **Variable** | | **Patients (n=20)** |
| --- | --- | --- |
| **Age (year, Mean ± SD)** | | 60.2 ± 12.11 |
| **Gender (n, %)** | **Male** | 14 (70.0) |
|  | **Female** | 6 (30.0) |
| **Stage**  **(n, %)** | **Stage I** | 10 (50.0) |
|  | **Stage II** | 3 (15.0) |
|  | **Stage III** | 7 (35.0) |
|  | **Stage IV** | 0 (0) |
| **T stage**  **(n, %)** | **T1** | 17 (85.0) |
|  | **T2** | 3 (15.0) |
|  | **T3** | 6 (30.0) |
|  | **T4** | 0 (0) |
| **N stage**  **(n, %)** | **N0** | 17 (85.0) |
|  | **N1** | 3 (15.0) |
| **M stage**  **(n, %)** | **M0** | 20 (100.0) |
|  | **M1** | 0 (0) |

**Supplementary Table 5. The characteristics of twenty ccRCC patients.**

**SD, Standard Deviation;**
