# Supplementary material for: Identification and Validation of a Ferroptosis-Related Long Non-Coding RNA (FRlncRNA) Signature to Predict Survival Outcomes and the Immune Microenvironment in Patients With Clear Cell Renal Cell Carcinoma
Source: Front Genet. 2022 Mar 8;13:787884. doi: 10.3389/fgene.2022.787884 (PMC8957844; doi:10.3389/fgene.2022.787884)
Supplement: Supplementary file 2 [file Table1.DOCX]

| **Variable** | | **Overall cohorts (n=507)** | **Training cohorts (n=243)** | **Testing cohorts (n=264)** | **ICGC cohorts**  **(n=89)** |
| --- | --- | --- | --- | --- | --- |
| **Age (year, Mean ± SD)** | | 60.26 ± 12.08 | 60.21 ± 12.46 | 60.31 ± 11.72 | 60.48 ± 10.06 |
| **Gender (n, %)** | **Male** | 333 (65.7) | 163 (67.1) | 170 (64.4) | 50 (56.2) |
|  | **Female** | 174 (34.3) | 80 (32.9) | 94 (35.6) | 39 (43.8) |
| **Stage**  **(n, %)** | **Stage I** | 253 (49.9) | 116 (47.7) | 137 (51.9) | 0 (0.0) |
|  | **Stage II** | 53 (10.5) | 28 (11.5) | 25 (9.5) | 0 (0.0) |
|  | **Stage III** | 116 (22.9) | 59 (24.3) | 57 (21.6) | 0 (0.0) |
|  | **Stage IV** | 82 (16.2) | 39 (16.0) | 43 (16.3) | 0 (0.0) |
|  | **Unknown** | 3 (0.5) | 1 (0.5) | 2 (0.7) | 89 (100.0) |
| **T stage**  **(n, %)** | **T1** | 259 (51.1) | 119 (49.0) | 140 (53.0) | 51 (57.3) |
|  | **T2** | 65 (12.8) | 34 (14.0) | 31 (11.7) | 9 (10.2) |
|  | **T3** | 172 (33.9) | 84 (34.6) | 88 (33.3) | 27 (30.3) |
|  | **T4** | 11 (2.2) | 6 (2.4) | 5 (2.0) | 2 (2.2) |
| **N stage**  **(n, %)** | **N0** | 225 (44.4) | 90 (37.0) | 135 (51.1) | 40 (44.9) |
|  | **N1** | 16 (3.2) | 10 (4.1) | 6 (2.3) | 0 |
|  | **NX** | 266 (52.4) | 143 (58.9) | 123 (46.6) | 49 (55.1) |
| **M stage**  **(n, %)** | **M0** | 401 (79.1) | 198 (81.5) | 203 (76.9) | 35 (39.3) |
|  | **M1** | 78 (15.4) | 38 (15.6) | 40 (15.2) | 4 (4.5) |
|  | **MX** | 26 (5.1) | 7 (2.9) | 19 (7.2) | 50 (56.2) |
|  | **Unknown** | 2 (0.4) | 0 (0.0) | 2 (0.7) | 0 (0.0) |
| **Survival status (n, %)** | **Alive** | 162 (34.0) | 77 (31.7) | 85 (32.2) | 60 (67.4) |
|  | **Dead** | 345 (66.0) | 166 (68.3) | 179 (67.8) | 29 (32.6) |
| **Survival years (Mean ± SD)** | | 3.25 ± 2.18 | 3.00 ± 2.03 | 3.48 ± 2.29 | 4.17 ± 1.69 |

**Table 1. The characteristics of ccRCC patients included in this study.**

**SD, Standard Deviation;**
