# Supplementary figures and images for: Identification and Validation of a Ferroptosis-Related Long Non-Coding RNA (FRlncRNA) Signature to Predict Survival Outcomes and the Immune Microenvironment in Patients With Clear Cell Renal Cell Carcinoma
Source: Front Genet. 2022 Mar 8;13:787884. doi: 10.3389/fgene.2022.787884 (PMC8957844; doi:10.3389/fgene.2022.787884)

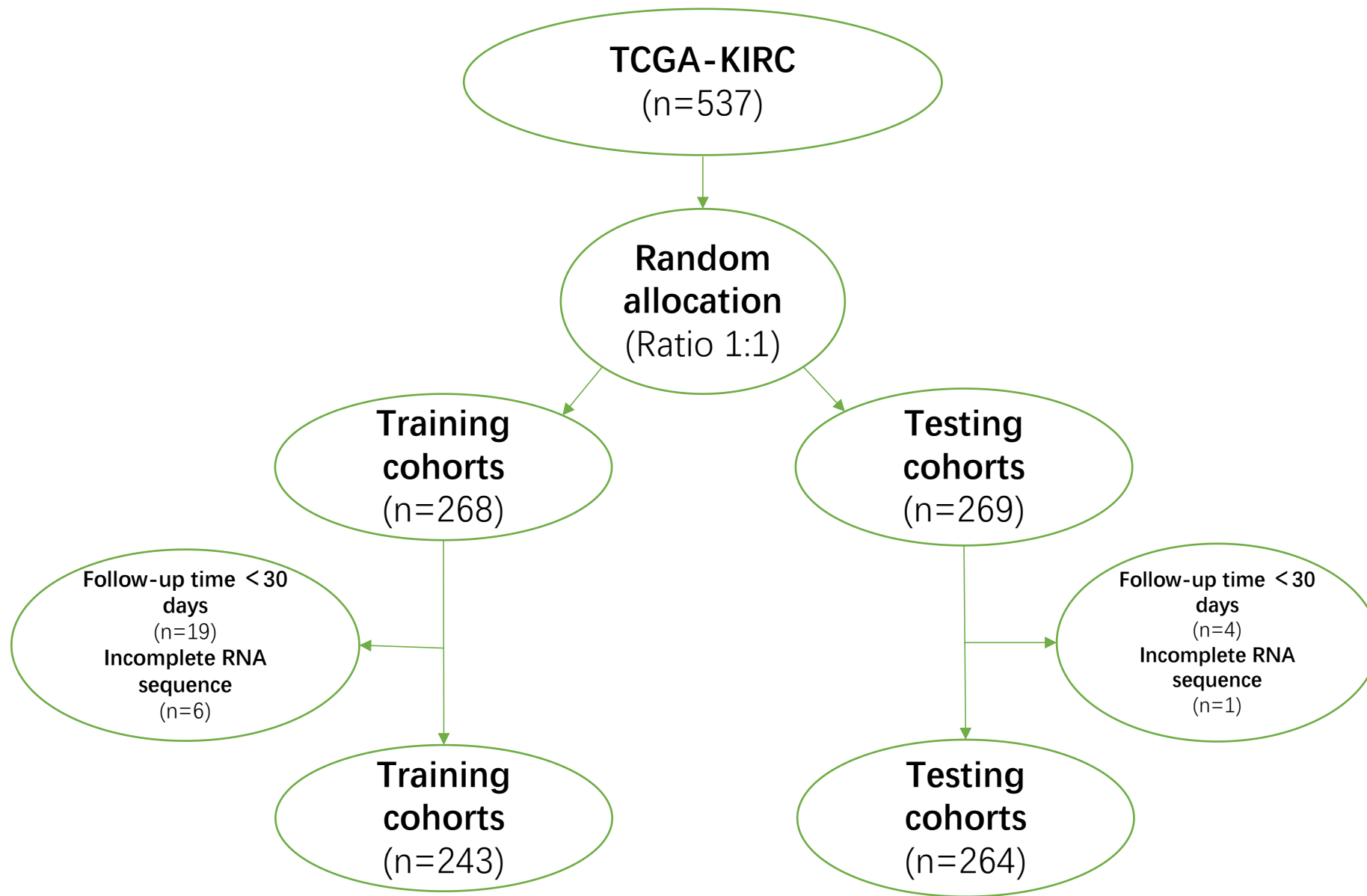

Supplement: Supplementary file 4 [file DataSheet2.ZIP › Supplementary figure/Supplementary Figure 1.pdf]

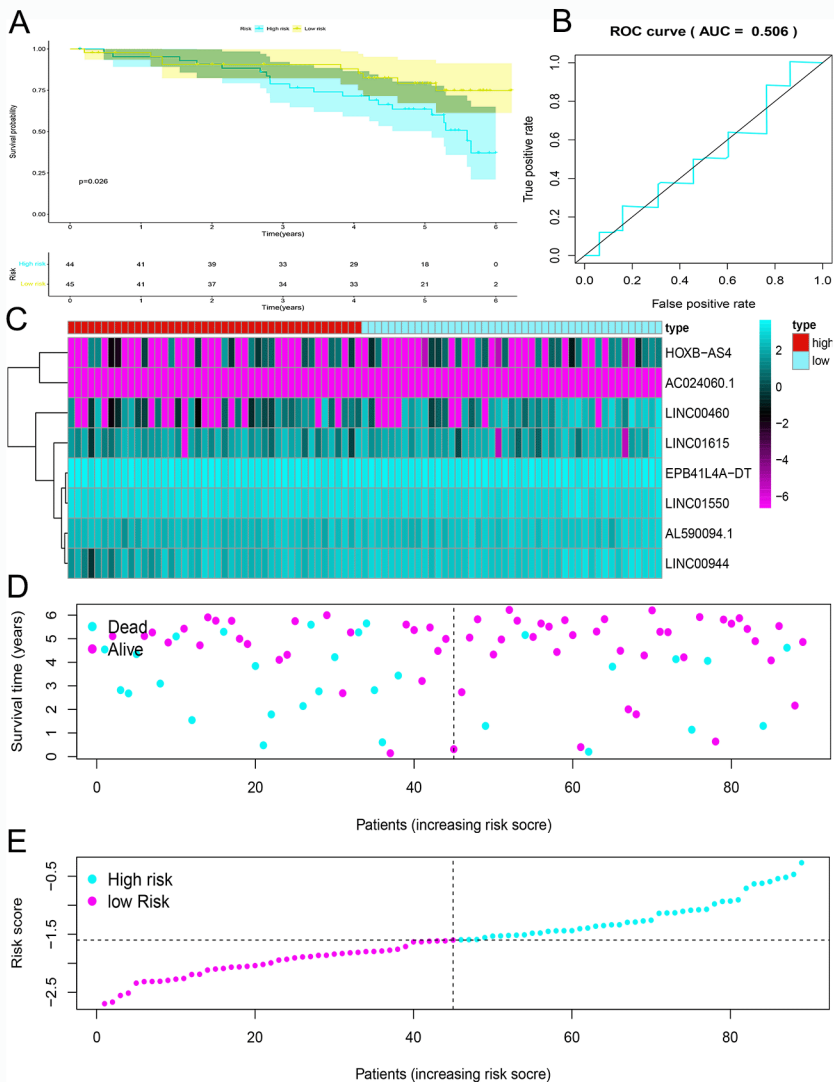

Supplement: Supplementary file 4 [file DataSheet2.ZIP › Supplementary figure/Supplementary Figure 2.pdf]

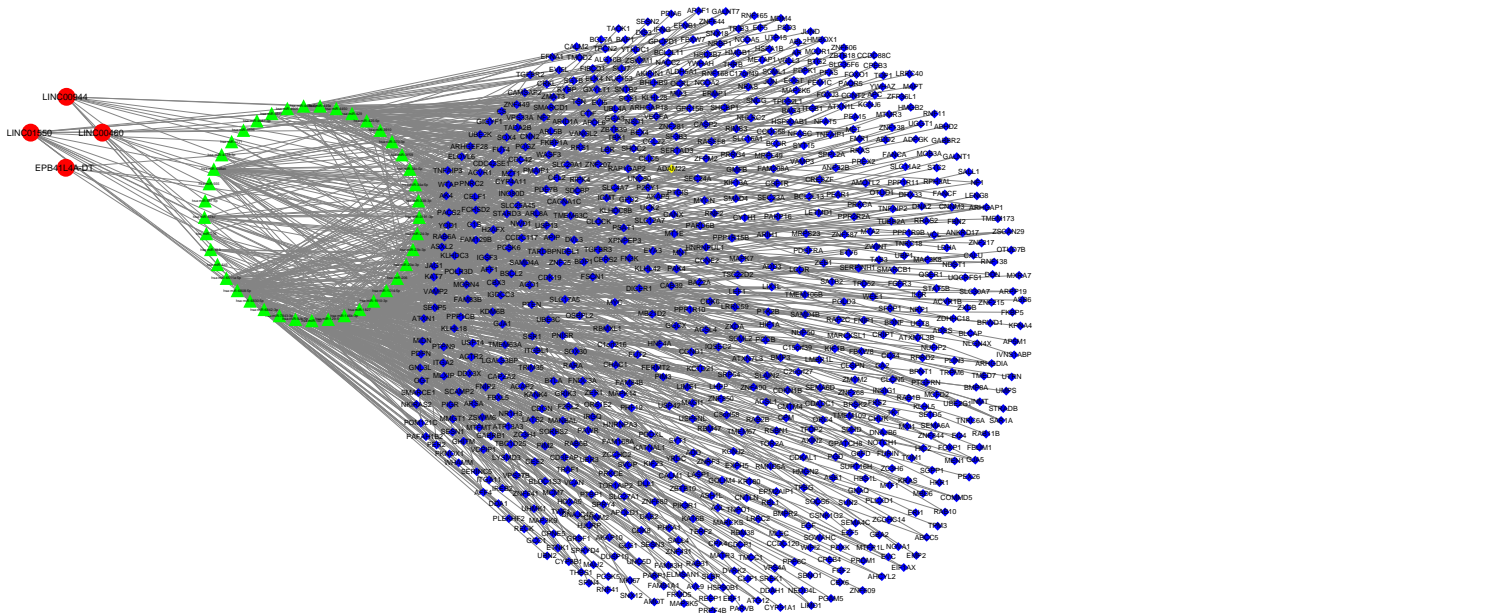

Supplement: Supplementary file 4 [file DataSheet2.ZIP › Supplementary figure/Supplementary Figure 3.pdf]
